# Two-photon fluorescence lifetime imaging of primed SNARE complexes in presynaptic terminals and β cells

**DOI:** 10.1038/ncomms9531

**Published:** 2015-10-06

**Authors:** Noriko Takahashi, Wakako Sawada, Jun Noguchi, Satoshi Watanabe, Hasan Ucar, Akiko Hayashi-Takagi, Sho Yagishita, Mitsuyo Ohno, Hiroshi Tokumaru, Haruo Kasai

**Affiliations:** 1 Faculty of Medicine, Laboratory of Structural Physiology, Center for Disease Biology and Integrative Medicine, University of Tokyo, Bunkyo-ku, Tokyo 113-0033, Japan; 2 CREST, Japan Science and Technology Agency, 4-1-8 Honcho, Kawaguchi, Saitama 332-0012, Japan; 3 PRESTO, Japan Science and Technology Agency, 4-1-8 Honcho, Kawaguchi, Saitama 332-0012, Japan; 4 Faculty of Pharmaceutical Sciences at Kagawa, Tokushima Bunri University, Kagawa 769-2193, Japan

## Abstract

It remains unclear how readiness for Ca^2+^-dependent exocytosis depends on varying degrees of SNARE complex assembly. Here we directly investigate the SNARE assembly using two-photon fluorescence lifetime imaging (FLIM) of Förster resonance energy transfer (FRET) between three pairs of neuronal SNAREs in presynaptic boutons and pancreatic β cells in the islets of Langerhans. These FRET probes functionally rescue their endogenous counterparts, supporting ultrafast exocytosis. We show that *trans*-SNARE complexes accumulated in the active zone, and estimate the number of complexes associated with each docked vesicle. In contrast, SNAREs were unassembled in resting state, and assembled only shortly prior to insulin exocytosis, which proceeds slowly. We thus demonstrate that distinct states of fusion readiness are associated with SNARE complex formation. Our FRET/FLIM approaches enable optical imaging of fusion readiness in both live and chemically fixed tissues.

## Abstract (web summary)

Synaptic vesicles are held in a fusion-competent state prior to their rapid release, which is thought to depend upon formation of *trans*-SNARE complexes. Takahashi *et al*. directly image this primed state using FLIM/FRET, and demonstrate differences in basal SNARE organization between neurons and β cells.

## Graphical abstract

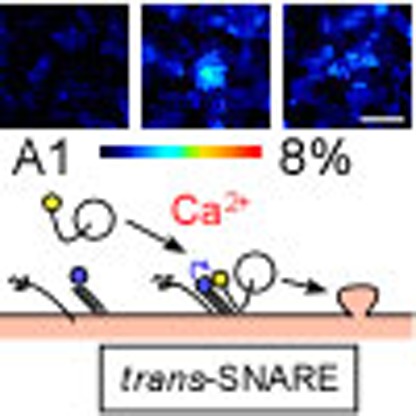

Ca^2+^-dependent exocytosis utilizes soluble *N*-ethylmaleimide-sensitive factor (NSF)-attachment protein receptors (SNAREs) for membrane fusion^1^. Three types of neuronal SNARE proteins—synaptosomal-associated protein 25 (SNAP25) and Syntaxin1A (Syx) in the plasma membrane (t-SNAREs) and vesicle-associated membrane protein 2 (VAMP2) in the vesicle membrane (v-SNARE)—assemble into α-helical four-helix bundles via their SNARE motifs through a zippering process that induces membrane fusion. It has been proposed that SNAREs partially assemble to form *trans*-SNARE complexes that connect the vesicle and plasma membranes and that Ca^2+^ triggers the completion of the zippering reaction, inducing exocytosis via Ca^2+^ sensors termed synaptotagmins and causing the formation of *cis*-SNARE complexes in the plasma membrane^1^,^2^.

This *trans*-SNARE mechanism is particularly suited to ultrafast exocytosis of synaptic vesicles in the active zone (AZ) of presynaptic terminals, where exocytosis is induced within a fraction of millisecond after increases in the cytosolic Ca^2+^ concentration^3^. No study, however, has ever succeeded in demonstrating the *trans*-SNARE complexes in AZ, because quantification of the assembly of the three proteins distinguishing their *trans*- and *cis*-states has been difficult in live cells^4^,^5^. The *trans*-SNARE complex may not be formed before stimulation^6^ in the slow secretory cells^5^,^7^, whereas the *trans*-SNARE mechanism is still assumed in these cells^8^,^9^. It has thus been largely conjectural how the kinetic diversity of exocytosis depends on the states of SNARE assembly in living cells.

We therefore established Förster resonance energy transfer (FRET) measurements between three pairs of SNARE proteins using t-SNAREs as FRET donors. We utilized two-photon fluorescence lifetime imaging (FLIM) and the time-correlated single photon counting method to monitor FRET^10^,^11^. FLIM utilizes only the donor fluorescence, and can estimate inter-molecular FRET, differentiating the FRET efficiency and the binding fraction of two proteins, unlike ratiometric FRET measurement^8^,^12^,^13^. SNAREs are the most abundant proteins in presynaptic boutons^14^, and are suitable for carrying out FRET analyses with least perturbation of endogenous proteins. We here confirmed that our green fluorescent protein-fused FRET proteins for SNAREs could replace endogenous proteins for ultrafast exocytosis using SNAP25 knockout mice. We established two independent (subtraction and gradient) methods to evaluate the fractions of *trans*-SNARE complexes in the presynaptic boutons of dissociated cultured cortical neurons. We applied the gradient method to hippocampal slice preparations, and found that *trans*-SNAREs were condensed in the AZ apposing dendritic spines. Moreover, the number of *trans*-SNAREs associated with each docked vesicle in AZ was estimated using the volume of dendritic spine as a proxy for AZ size^15^. In a striking contrast, *trans*-SNAREs were absent in the resting state, and formed only after stimulation in the plasma membrane of β cells in the pancreatic islets of Langerhans, which undergo slow exocytosis of insulin (>1 s)^12^,^16^,^17^. Thus, we have established an approach to image fusion readiness in various secretory preparations.

## Results

### Estimation of binding fractions of SNAREs with FRET/FLIM

**Figure 1:**
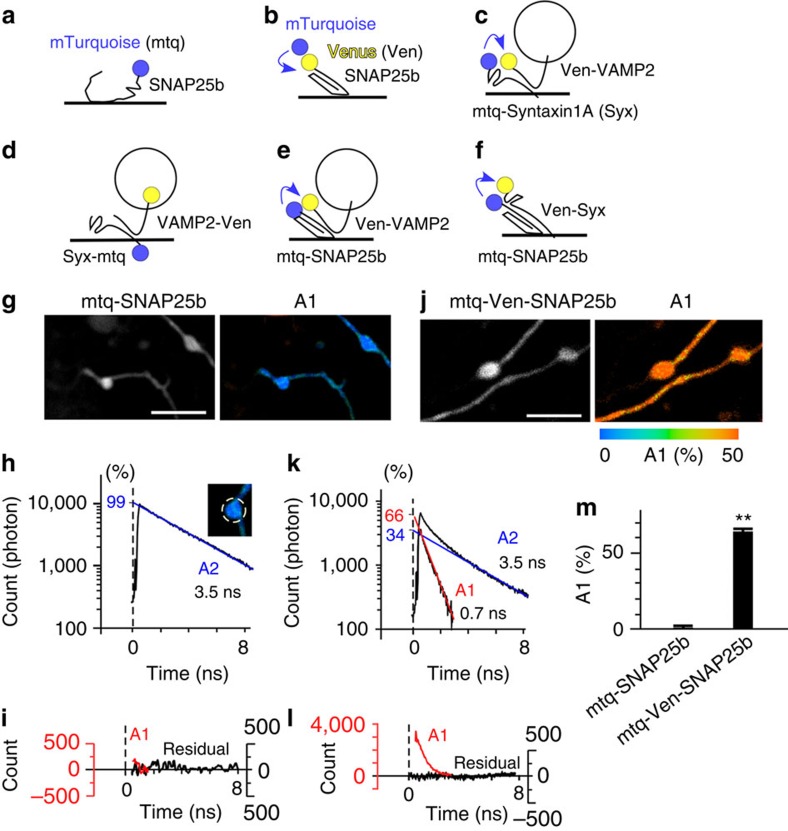
Combinations of FRET/2pFLIM probes for SNAREs. (**a**–**f**) Schematic drawing of combinations of probes whose constructs are depicted in Supplementary Fig. 1. mTurquoise was used as a donor and Venus as an acceptor. The N terminal end of synaptosomal-associated protein 25 (SNAP25b) was labelled with either mTurquoise (mtq, **a**) or in tandem with mtq and Venus (Ven, **b**) to measure the folding fraction of Ven. The binding fractions (or A1 fractions) of syntaxin1A (Syx) with vesicle-associated membrane protein 2 (VAMP2) in the cytosol were detected by mtq-Syx and Ven-VAMP2 (**c**). The *cis*-complex of Syx with VAMP2 was detected by Syx-mtq and VAMP2-Ven (**d**), because FRET occurs only when they are present in the same membrane. The assembly of SNAP25 with VAMP2 or Syx was detected by mtq-SNAP25 and Ven-VAMP2 (**e**) or Ven-Syx (**f**). (**g**) Images of synaptic boutons in rat cortical dissociated culture lipofected with mtq-SNAP25 (scale bar, 5 μm). The left image shows the fluorescence intensity of mtq-SNAP25, while the right image shows the fluorescence decay of the A1 fraction for each pixel in pseudocolour coding shown in **j**. (**h**) A lifetime decay curve in a semi-logarithmic plot, which was obtained from eight boutons. The decay fits well with a single exponential function with a time constant of 3.5 ns. (**i**) The residual data after subtracting the exponential function. (**j**) Images of synaptic boutons lipofected with the tandem mtq-Ven-SNAP25b probe (scale bar, 5 μm). (**k**) A lifetime decay curve in a semi-logarithmic plot obtained from 13 boutons, which fits well with a double-exponential function, with time constants of 0.7 ns (*τ*_1_) and 3.5 ns (*τ*_2_) and weights of 66% (A1) and 34% (A2), respectively. (**l**) The residual data after subtracting the A2 component (red) or both components (black). (**m**) The A1 fractions of mtq-SNAP25 (mean±s.e.m.=0.28±1.9%; 19 boutons) and mtq-Ven-SNAP25 probes (63.5±1.2%; 12 boutons). Wilcoxon signed-rank test versus 0%: *P*=0.71 and **P=0.002, respectively.

To quantify assembly between the three pairs of SNARE proteins, we labelled the t-SNAREs SNAP25b and Syx with mTurquoise (mtq) to form FRET donors and VAMP2 and Syx with the FRET acceptor Venus (Ven; Fig. 1a–f and Supplementary Fig. 1). The constructs were lipofected into cortical neurons in dissociated culture. Images were acquired for 60 s to accumulate a sufficient number of photons for constructing fluorescence lifetime curves.

The fluorescence lifetime of mtq-SNAP25b (Fig. 1a) was monoexponential, with a time constant of ∼3.5 ns, in synaptic boutons (Fig. 1g–i; A2), indicating that mtq is an appropriate probe for quantitative FLIM^18^,^19^. A faster decay component (A1), with time constants of 0.7 ns (*τ*_1_), was evident when both mtq and Ven were fused with SNAP25b in tandem (Fig. 1b,j–l; A1 (ref. 20)). The fast component fraction (A1) was obtained by double-exponential fitting of photon count decays by fixing the slow component as 3.5 ns (*τ*_2_; Methods). The A1 fraction was estimated to be 63.5% (Fig. 1m) instead of 100% that would be expected for a tandem probe. This is in line with a previous study^10^, and is explained by incomplete maturation or a dark state of Ven. This factor was left uncorrected to avoid possible overestimation of molecular interactions^10^.

**Figure 2:**
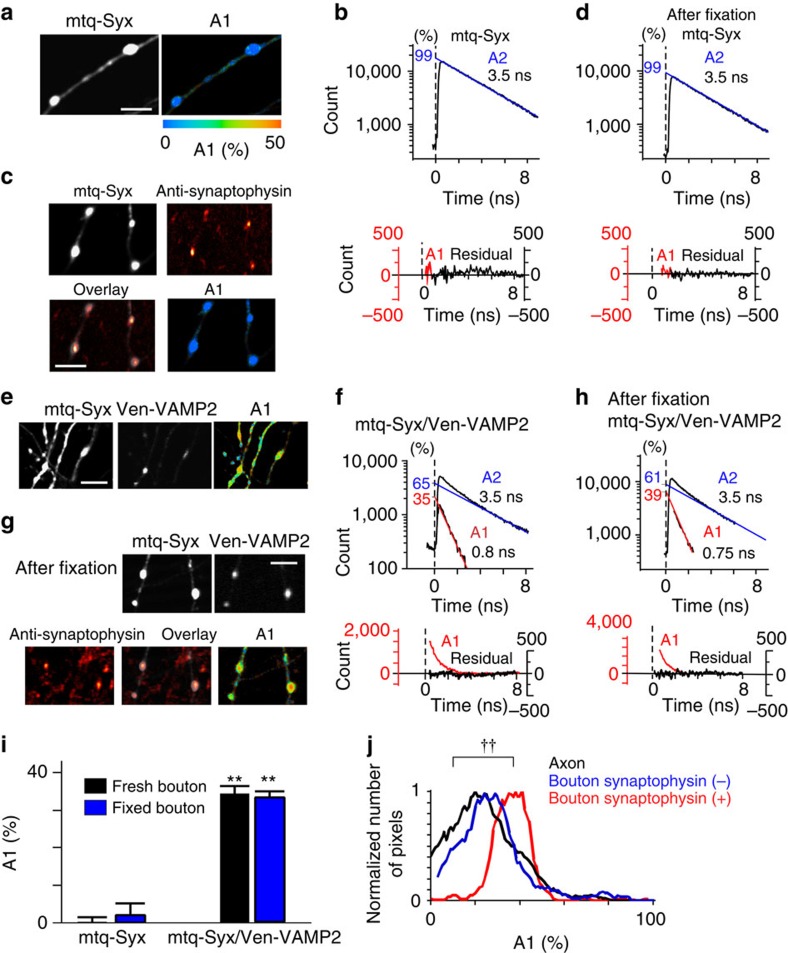
FRET/2pFLIM of the SNARE complexes between mtq-Syx and Ven-VAMP2 in neurons. (**a**,**c**) Fluorescence and A1 fraction images of mtq-Syx-expressing boutons (scale bars, 5 μm). Preparations were live-imaged in **a** and were imaged after chemical fixation and counterstaining with anti-synaptophysin antibody in **c**. (**b**,**d**) Lifetime decay curves of mtq-Syx obtained from 14 boutons. (**e**,**g**) Fluorescence and A1 fraction images of mtq-Syx/Ven-VAMP2-stained boutons (scale bars, 5 μm). Preparations were live-imaged in **e** and were imaged after chemical fixation and counterstaining with anti-synaptophysin antibody in **g**. (**f**,**h**) Lifetime decay curves of mtq-Syx/Ven-VAMP2 obtained from 12 live boutons and 8 fixed boutons. (**i**) The A1 fractions of mtq-Syx (black bar; mean±s.e.m.=0.18±1.4%; 14 boutons) or after fixation (blue bar; 2.4±2.9%; 14 boutons) and mtq-Syx/ven-VAMP2-stained boutons in live images (black bar; 34.1±2.0%; 21 boutons) and after fixation (blue bar; 33.2±1.3%; 19 boutons) at the endogenous expression level of VAMP2 (Supplementary Fig. 3b). The A1 fractions for mtq-Syx/Ven-VAMP2 were significantly different from 0% (**P=0.0004 and **P=0.0001 using the Wilcoxon signed-rank test). The A1 fraction was not affected by fixation (*P*=0.74 using the Mann–Whitney *U*-test). (**j**) Distributions of the A1 fractions in the boutons among the pixels positive (red; 38.8±1.8%; 519 pixels) or negative (blue; 17.4±2.2%; 323 pixels) for anti-synaptophysin staining or in axons (black; 15.6±2%; 183 pixels; *P*=0.0002 using the Kruskal–Wallis test; *P*=0.75 and ^††^*P*=0.0039 for boutons negative and positive for synaptophysin versus axons using Steel's test).

**Figure 3:**
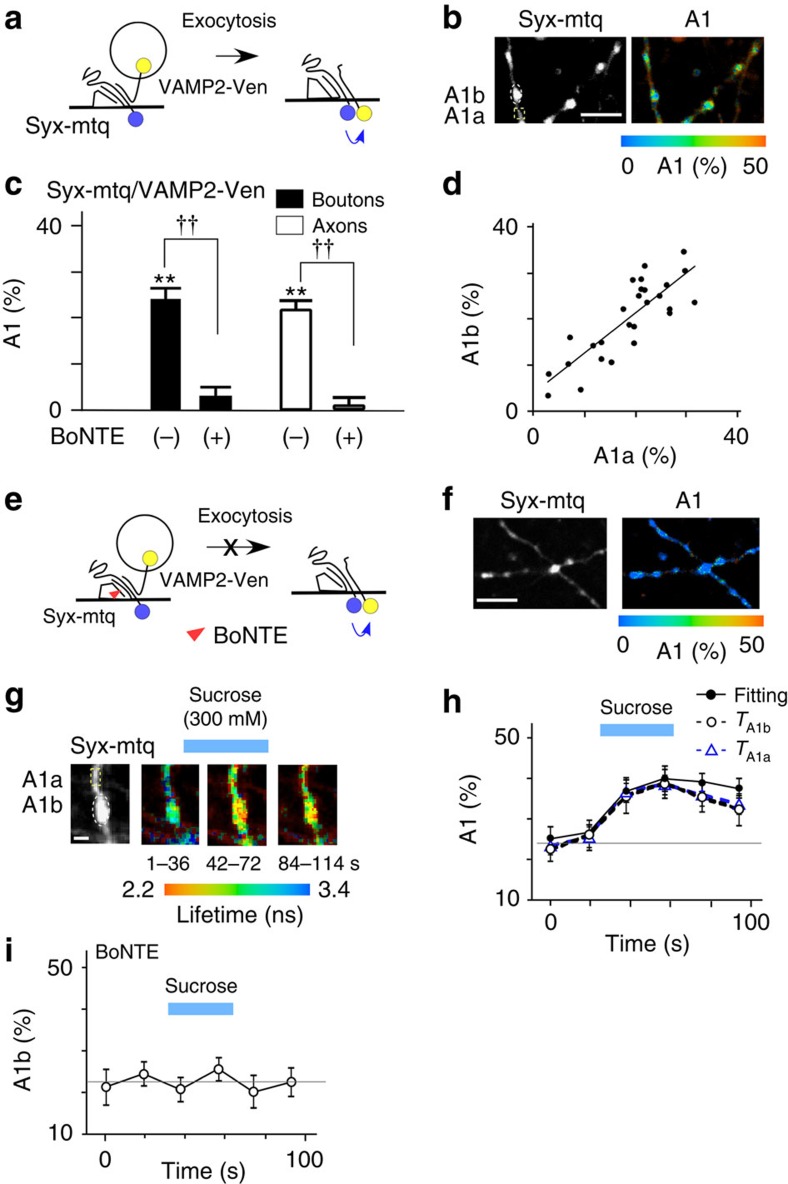
FRET/2pFLIM of *cis*-SNARE complexes between Syx-mtq and VAMP2-Ven in neurons. (**a**) Schematic drawing of the conformation change of the *cis*-probes via exocytosis. (**b**) Fluorescence and A1 fraction images of Syx-mtq/VAMP2-Ven-expressing boutons (scale bar, 5 μm). (**c**) The average A1 fractions of Syx-mtq/VAMP2-Ven-stained boutons (black) and axons (open) in the absence and presence of the light chain of botulinum toxin (BoNTE). The A1 fractions in boutons (24.7±2.1%; 24 boutons) and axons (21.5±2.0%; 19 axons) were significantly different from 0% (**P=0.0001 and 0.00001 using the Wilcoxon signed-rank test) in the absence of BoNTE at the mean expression levels of VAMP2-Ven of 524±45 a.u. and 508±26 a.u., respectively (Supplementary Fig. 3d). They were not significantly different from 0% in the boutons (3.5±2.1%; 22 boutons) and axons (0.9±2%; 12 axons) in the presence of BoNTE using the Wilcoxon signed-rank test (*P*=0.12 and 0.53). The Mann–Whitney *U*-test yielded ^††^*P*=0.00001 for boutons without and with toxin and ^††^*P*=0.00001 for axons. (**d**) The correlation between the A1 fractions in the boutons (A1b) and those of axons surrounding the boutons (A1a; *P*=0.0001 using Spearman's rank correlation coefficient; *r*=0.80; *n*=27). (**e**) Schematic drawing of the *cis*-configuration and the site of BoNTE action. (**f**) Fluorescence and A1 fraction images of boutons expressing Syx-mtq, VAMP2-Ven and BoNTE (scale bar, 5 μm). (**g**) Fluorescence lifetime images of Syx-mtq/VAMP2-Ven-stained boutons during 300 mM sucrose stimulation (scale bar, 1 μm). (**h**) The time courses of the A1 fractions estimated either by decay curve fitting (filled circles, 21 boutons) or average lifetimes (open symbols, 21 boutons) in the boutons (*T*_A1b_, open circles, *n*=19) and axons surrounding the boutons (*T*_A1a_, open triangles). All values after sucrose stimulation were significantly (*P*<0.01) different from those before stimulation. (**i**) The time courses of A1b estimated by average lifetimes during 300 mM sucrose stimulation in the preparation treated with BoNTE (*n*=19).

**Figure 4:**
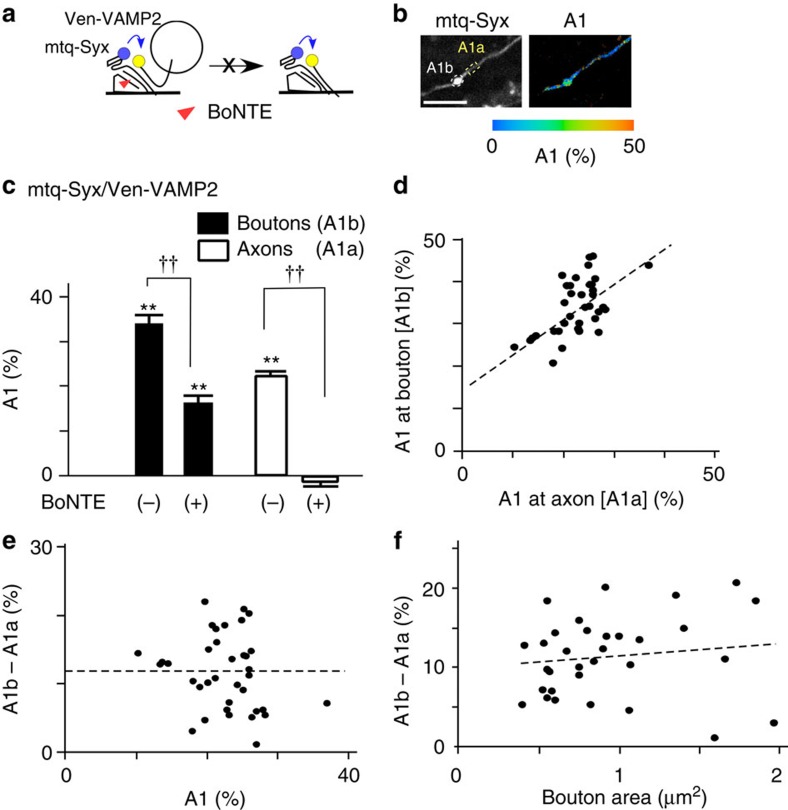
Action of the light chain of botulinum toxin E (BoNTE) on the SNARE complexes. (**a**) Schematic drawing of the *trans*-complex with mtq-Syx/Ven-VAMP2 and the site of action of the light chain of BoNTE, which blocks exocytosis and the formation of the *cis*-complex. (**b**) Fluorescence and A1 fraction images of boutons simultaneously lipofected with mtq-Syx, Ven-VAMP2 and BoNTE (scale bar, 5 μm). (**c**) The A1 fractions of boutons in the absence (the same data as Fig. 2i) and presence of BoNTE (16.3±1.6%, 39 boutons) at the endogenous expression level of VAMP2 (Supplementary Fig. 3b) and of axons in the absence (open bars, 22.4±0.9%; 33 axons) and in the presence of BoNTE (−1.9±1.8%; 17 axons). Wilcoxon signed-rank test versus 0%: **P=0.0001 in BoNTE-treated boutons, whereas **P=0.0003 and *P*=0.42 in axons untreated or treated with BoNTE. The A1 fraction values of BoNTE versus control in boutons and axons differed with ^††^*P*=0.0006 and ^††^*P*=0.004 using the Mann–Whitney *U*-test, respectively. (**d**) The correlation between the A1 fractions of individual boutons (A1b) and those in the surrounding axons (A1a; *P*=0.0012 using Spearman's rank correlation coefficient; *r*=0.59; *n*=36). (**e**) The correlation between (A1b–A1a) and A1a (*P*=0.21 using Spearman's rank correlation coefficient; *r*=−0.23, *n*=36). (**f**) The correlation between (A1b–A1a) and the bouton area in its central Z-section (*P*=0.20 using Spearman's rank correlation coefficient; *r*=0.13, *n*=32).

**Figure 5:**
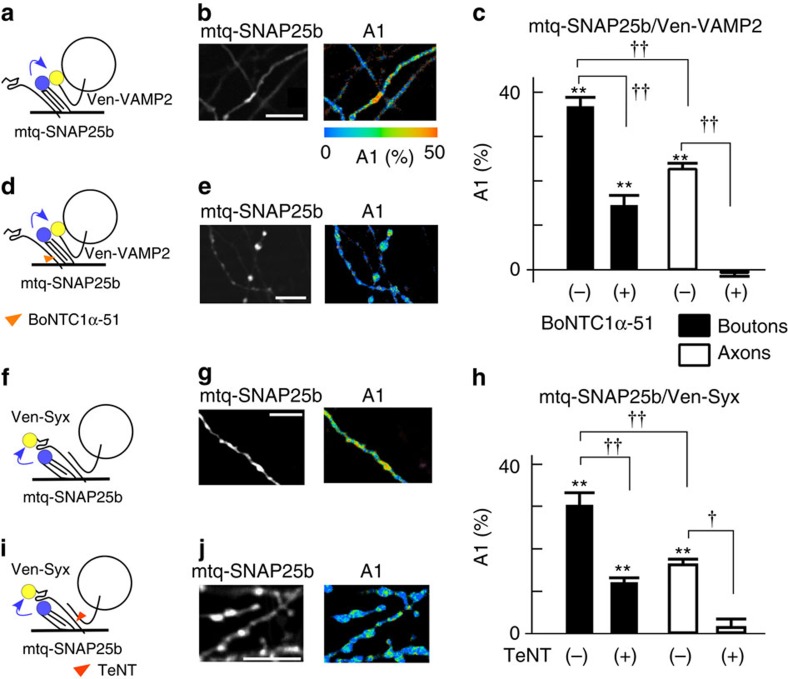
FRET/2pFLIM of mtq-SNAP25-based probes for SNARE complexes. (**a**,**d**) Schematic drawing of mtq-SNAP25/Venus-vesicle-associated membrane protein 2 (Ven-VAMP2) probes and the action of BoNTC1α-51. (**b**,**e**) Fluorescence and A1 fraction images of mtq-SNAP25/Ven-VAMP2-expressing boutons in the absence (**b**) and presence of BoNTC1α-51 (**e**) (scale bars, 5 μm). (**c**) The A1 fractions of mtq-SNAP25/Ven-VAMP2-expressing boutons (filled; 36.7±2.2%; 20 boutons at the endogenous expression level of VAMP2; Supplementary Fig. 3f) and axons (open; 22.7±1.3%; 22 axons) and those in the presence of BoNTC1α-51 in boutons (14.5±2.2%; 24 boutons) and in axons (−0.4±1.1%; 17 axons). **P<0.001 for control boutons and axons and **P=0.0001 for BoNTC1α-51-treated boutons using the Wilcoxon signed-rank test versus 0%. Using the Kruskal–Wallis test on the four groups, we obtained a *P* value of <0.00001, and the effects of BoNTC1α-51 on boutons and axons were †*P*=0.03 and †*P*=0.04, respectively, using Scheffé's test. ††*P*=0.004 between control boutons and axons and ^††^*P*=0.002 between BoNTC1α-51-treated boutons and axons. (**f**,**i**) Schematic drawing of mtq-SNAP25/Ven-Syx probes and the action of TeNT. (**g**,**j**) Fluorescence and A1 fraction images of mtq-SNAP25/Ven-Syx-expressing boutons in the absence (**g**) and presence of tetanus toxin (TeNT; **j**) (scale bars, 5 μm). (**h**) The average A1 fractions of mtq-SNAP25/Ven-Syx-stained boutons (filled; mean±s.e.=29.5±3.1%; 21 boutons with the mean expression of Ven-Syx of 139 a.u.; Supplementary Fig. 3h) and axons (open; 16.2±1.3%; 28 axons), those of boutons in the presence of TeNT (12.0±1.1%; 45 boutons with the mean expression level Ven-Syx of 138 a.u.) and of axons (2.4±1.7%; 15 axons). **P<0.01 for control boutons, axons and TeNT-treated boutons using the Wilcoxon signed-rank test versus 0%. Using the Kruskal–Wallis test on the four groups, we obtained a *P* value of <0.00001. The A1 values of boutons and axons were different (^††^*P*=0.004) in the absence of the toxin, and the toxin's effects were significant: ^††^*P*=0.0007 and †*P*=0.04 for boutons and axons using Scheffé's test.

**Figure 6:**
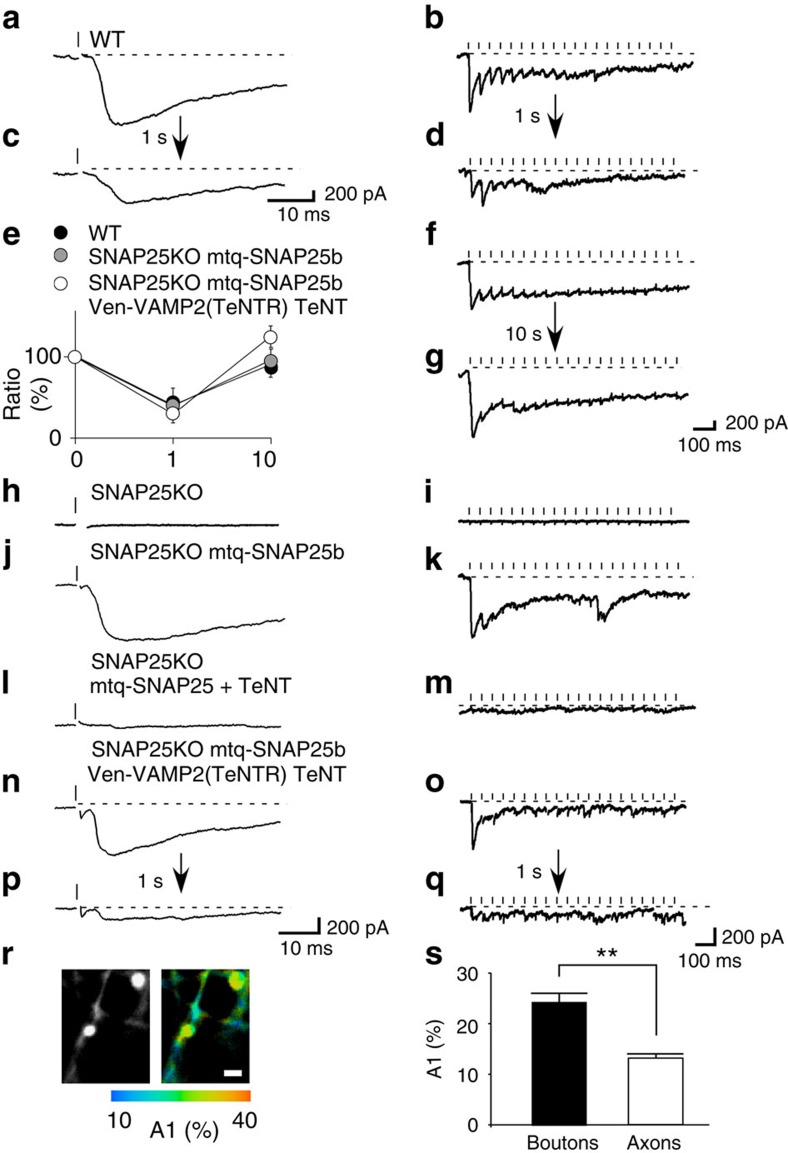
Kinetics of EPSCs, depletion and refilling of vesicles mediated by mtq-SNAP25b/Ven-VAMP2 in SNAP25 KO mice treated with TeNT. (**a**,**b**) EPSCs recorded from dissociated cultures from wild-type (WT) mice after the first stimulation (**a**) and during 20 repetitive stimuli at 20 Hz in the presence of picrotoxin (50 μM) and cyclothiazide (100 μM). The current traces were recorded with whole-cell patch clamp, and presynaptic fibres were stimulated with a glass electrode. (**c**,**d**) EPSCs evoked from the same cell by the second repetitive stimulation 1 s after the first train of stimuli. (**e**) Amplitudes of the first EPSCs in the repetitive stimulation relative to that of the first train of stimulation. Two trains were separated by 1 s in the preparations from WT mice (filled circles, 40±21%, *n*=6, *P*=0.04), those from the KO mice rescued with mtq-SNAP25b (grey circles, 29±8.5%, *n*=5, *P*=0.043), and those from the KO mice expressing mtq-SNAP25b and Ven-VAMP2 (TeNT resistant) after treatment with TeNT (open circles, 39±12%, *n*=8, *P*=0.01). The *P* values were obtained with the Wilcoxon signed-rank test. The amplitudes recovered to 91±16% (*n*=6, *P*=0.6), 96±13% (*n*=4, *P*=0.1), and 125±13% (*n*=4, *P*=0.9) when the interval was set to 10 s. (**f**,**g**) EPSCs evoked by the first (**f**) and second (**g**) repetitive stimulation separated by 10 s. (**h**–**m**) EPSCs recorded from dissociated cultures obtained from SNAP25 KO mice (**h**,**i**), those rescued by mtq-SNAP25b (**j**,**k**) and those with exocytosis blocked by TeNT treatment (**l**,**m**). (**n**–**q**) Current traces recorded from dissociated culture preparations from SNAP25 KO mice expressing mtq-SNAP25 and Ven-VAMP2(TeNTR), and treated with TeNT for 1 day. The first and second repetitive stimuli were separated by 1 s. (**r**) Fluorescence and A1 images of mtq-SNAP25b/Ven-VAMP2(TeNTR)-expressing boutons (scale bar, 1 μm). (**s**) The mean A1 values in boutons (24.4±1.6%, *n*=23) and axons (13.1±0.7%, *n*=35). A Mann–Whitney *U*-test yielded a **P value of 0.0000. The average expression levels of mtq-SNAP25 and Ven-VAMP2(TeNTR) were 79±6.7 a.u. and 541±39 a.u., respectively.

**Figure 7:**
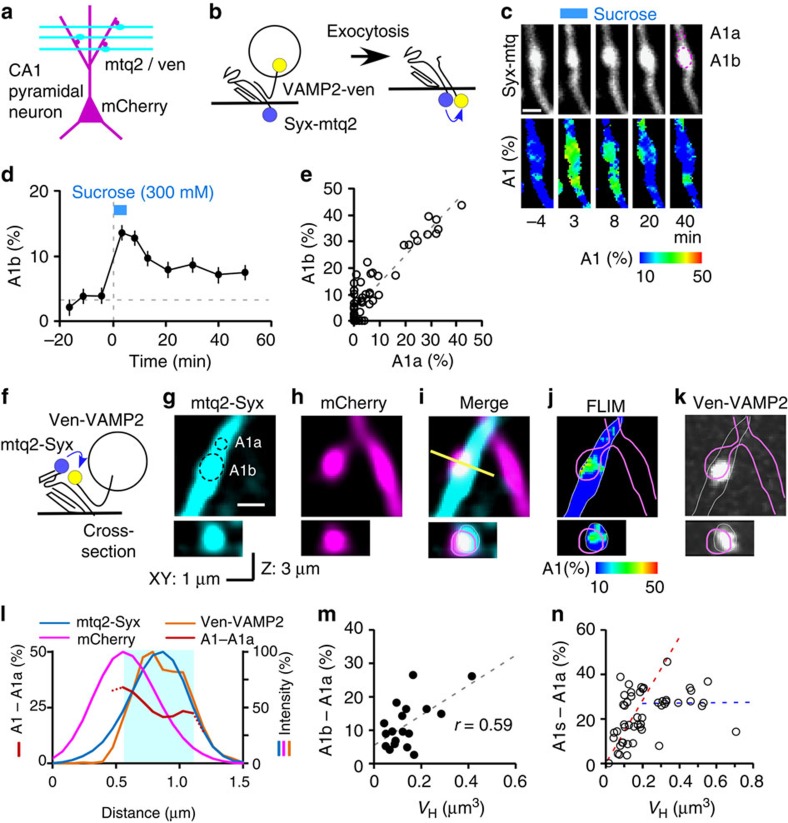
FRET/2pFLIM of presynaptic boutons on the dendritic spines of CA1 pyramidal neurons in slice culture preparations. (**a**) Schematic drawing of Schaffer collateral fibres on CA1 pyramidal neurons in the stratum radiatum. (**b**) Schematic drawing of *cis*-SNARE probes, syntaxin1A-mtq2 (Syx-mtq2) and VAMP2-Ven, which induce FRET after exocytosis. (**c**) Fluorescence lifetime images of Syx-mtq2/VAMP2-Ven-stained boutons during 300 mM sucrose stimulation (scale bar, 1 μm). (**d**) Average time courses of the A1 fraction in boutons during sucrose application (24 boutons, 8 axons). (**e**) The correlation between the A1 fractions in the boutons (A1b) and those of axons surrounding the boutons (A1a; *P*<0.0001 in Spearman's rank correlation coefficient; *r*=0.74; *n*=59). (**f**) Schematic drawing of the SNARE probes mtq2-Syx and Ven-VAMP2 in the *trans*-complex. (**g**,**h**) Fluorescence images (Z-stacks of three sections) of a Schaffer collateral axon expressing mtq2-Syx (**g**) and Ven-VAMP2, which were transfected into CA3 neurons using an AAV1, and of dendritic spines expressing mCherry (**h**) expressed by gene gun (scale bar, 1 μm). These images were simultaneously acquired with the same excitation laser at 900 nm. (**i**) The overlap of a bouton (**g**) and spine (**h**) in both the *xy* plane (upper panel) and *xz* plane along the yellow line (lower panel). (**j**) A lifetime image of mtq2 obtained from the image (**g**), which shows a high FRET region at the edge of the axonal bouton facing the spine in both *xy* and *xz* images. (**k**) A fluorescence image of Ven-VAMP2 obtained from the same area at 980 nm, where the precise location of Ven-VAMP2 relative to the dendrite was aligned by simultaneous imaging of Ven-VAMP2 and mCherry. (**l**) The fluorescence profiles along the yellow line in **i** for mtq2-Syx, Ven-VAMP2, mCherry and A1-A1a fractions of mtq lifetime images. The cyan-shaded region represents the axonal area where the fluorescent intensity of mtq-Syx was 50% of the peak value. (**m**) The correlation between spine volume (*V*_H_) and A1b–A1a. Spearman's rank correlation coefficients were *r*=0.59 (*P*=0.008, *n*=19). (**n**) The correlation between spine volume (*V*_H_) and the peak A1b value at the point facing the spine (A1s–A1a, **n**). In addition to the data points in (**m**), we intentionally sampled more spines from larger spines. Spearman's rank correlations was 0.45 (*P*=0.0095, *n*=32, the red dashed line) for spines with volumes <0.2 μm^2^, while −0.067 (*P*=0.86, *n*=18, the blue horizontal dashed line) for spines with volume >0.2 μm^2^. The average A1s–A1a values for spines with volumes >0.25 μm^2^ was 27.7±2.1% (*n*=16).

**Figure 8:**
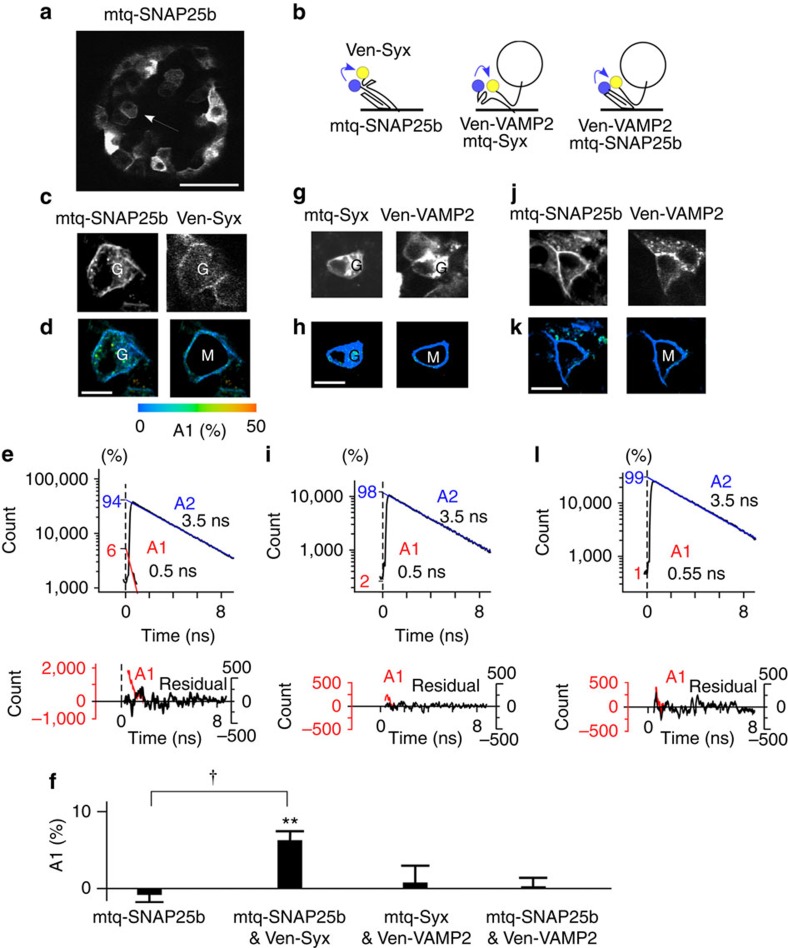
FRET/2pFLIM with SNARE probes in the β cells in the pancreatic islets of Langerhans. (**a**) A fluorescence image of islets transfected with mtq-SNAP25 by lentivirus (scale bar, 50 μm). The arrow indicates the typical cells investigated. (**b**) Schematic drawings of three FRET probes. (**c**,**d**,**g**,**h**,**j**,**k**) Fluorescence and A1 fraction images of mtq-SNAP25/Venus-syntaxin (Ven-Syx; **c**,**d**), mtq-Syx/Ven-VAMP2 (**g**,**h**) or mtq-SNAP25 and Ven-VAMP2 (**j**,**k**) (scale bars, 5 μm). ‘G' denotes the Golgi apparatus, and ‘M' in the right panels of images (**d**,**h**,**k**) denotes the masked cytosolic regions. (**e**,**i**,**l**) Lifetime decay curves of the plasma membranes of β cells transfected with mtq-SNAP25/Ven-Syx (**b**, **e**), mtq-Syx/Ven-VAMP2 (**b**, **i**), and mtq-SNAP25 and Ven-VAMP2 (**b**, **l**), which were obtained from 12, 12 and 11 cells, respectively. (**f**) The average A1 fractions of the lifetime decay of β-cell plasma membrane transfected with four different probes indicated. The A1 fractions of mtq-SNAP25 are −0.8±1.1% (10 cells; *P*=0.14), those mtq-SNAP25/Ven-Syx are 5.3±1% (22 cells; **P<0.01), those of mtq-Syx/Ven-VAMP2 are 0.8±2.3% (15 cells; *P*=0.82) and those of mtq-SNAP25/Ven-VAMP2 are 0.04±1.4% (21 cells; *P*=0.89) at the endogenous expression levels of Ven-Syx and Ven-VAMP2 (Supplementary Figs 3f,h and 8d,f), respectively. The *P* values were obtained using the Wilcoxon signed-rank test. The four groups were significant in analysis using the Kruskal–Wallis test with *P*<0.007. Steel's test versus mtq-SNAP25 yielded *P*=0.014 for mtq-SNAP25/Ven-Syx, *P*=0.92 for mtq-Syx/Ven-VAMP2 and *P*=0.74 for mtq-SNAP25/Ven-VAMP2.

The values of *τ*_1_ for all the probes (Fig. 1 and Figs 2, 3, 4, 5, 6, 7, 8) were estimated in between 0.5 and 1.5 ns, indicating that FRET efficiency (=1—*τ*_1_/*τ*_2_) was between 0.85 and 0.57 and that distances between the two fluorescent proteins in the binding state were within the Förster radius, and therefore the A1 value was used as an estimate of the binding fraction (%) of a SNARE pair. We first analysed FRET probes in neurons in dissociated culture and then applied them to intact synapses in slice preparations, as we are using two-photon imaging, then compared the results with those of β cells in the islets of Langerhans.

### Syx-based FRET probes in synaptic boutons

The time constant of the fluorescence decay of mtq-Syx was monoexponential and unaffected by chemical fixation (Fig. 2a–d,i). We confirmed that Syx-positive boutons were presynaptic structures by counterstaining mtq-Syx preparations with anti-synaptophysin antibody (Fig. 2c). There was no gradient of A1 values within a single bouton around the synaptophysin-positive spots (Fig. 2c; A1). However, significant A1 fractions appeared within a bouton (Fig. 2e,f) when Ven-VAMP2 was cotransfected with mtq-Syx. We confirmed that the A1 fraction represented FRET, because it was eliminated by bleaching of Ven (Supplementary Fig. 2a,b). Comparable A1 fractions were obtained when we used the monomerizing A206K mutant of Ven (mVen; Supplementary Fig. 2c,d), in which dimerization of Ven and binding between mtq and Ven were minimized^21^.

The A1 values did not seem to depend on expression levels of the donor mtq-Syx, but correlated with the expression of the acceptor Ven-VAMP2 (Supplementary Fig. 3a,b). Endogenous expression of VAMP2 was estimated using an antibody against VAMP2 as 540 a.u. (Supplementary Fig. 4a; Methods), where the specificity of the antibodies to VAMP2 was confirmed by transfecting the light chains of TeNT, which selectively cleaves VAMP2, and removed 90% of antigenicity (Supplementary Fig. 5a,b). The A1 fraction was 34.1% in boutons (Fig. 2i) when expression levels of VAMP2 were similar to endogenous levels (Supplementary Fig. 3b). This A1 fraction should predict the binding fraction of endogenous Syx with VAMP2, given that the FRET signal showed an approximately linear increase with VAMP2 expression levels (Supplementary Fig. 3b). The A1 fraction was not ascribed to molecular crowding of SNAREs in AZ because the homotypic binding between mtq-Syx and Ven-Syx (Supplementary Fig. 6a–e), and between mtq-SNAP25 and Ven-SNAP25 (Supplementary Fig. 6e–i), were negligible at all expression levels, whereas all three heteromeric pairs of SNAREs (Syx/VAMP2, SNAP25/VAMP2 and SNAP25/Syx) showed significant binding (Fig. 2; see Fig. 5).

Chemical fixation did not affect fluorescence lifetimes^22^ and FRET values (Fig. 2g,h), and we found that synaptophysin-positive sites in boutons showed FRET in the A1 fraction of 38.8%, whereas the synaptophysin-negative parts showed FRET in the A1 fraction of 17.4% (Fig. 2j; see Fig. 7j–l for more precise arrangement of vesicles and the high FRET region). This suggests that the Syx- and VAMP2-binding fractions were larger in the AZ. In contrast, the A1 fraction was only ∼22% in axons on average (Fig. 2j; Fig. 4c). These binding fractions included both *trans*- and *cis*-SNARE complexes.

To quantify *cis*-SNARE complex separately from *trans*-complex^1^, we labelled SNAREs at their C-termini so that FRET occurred only for *cis*-SNARE complexes (Fig. 3a). A1 values were estimated to be 24.7% at the endogenous level of VAMP2 expression (Fig. 3b–d and Supplementary Fig. 3d). Since the total fraction of SNARE complexes was estimated as 34.1% (Fig. 2i), this provided an estimate of the fraction of Syx that forms *trans*-SNARE complexes with VAMP2 of 9.4% (=34.1−24.7) on average (Supplementary Table 1; subtraction method). Unlike the A1 fractions of total SNARE complexes (Fig. 2j), those of *cis*-SNAREs were similar between boutons and axons (Fig. 3c; 24.7 versus 21.5%). Moreover, the A1 fractions in the boutons (A1b) were similar to those in the axons (A1a) surrounding each bouton (Fig. 3d), suggesting that *cis*-SNAREs are diffusible and equilibrate along axons. Furthermore, *cis*-complex was nearly eliminated by lipofection of the light chain of botulinum toxin, BoNTE, which selectively cleaves SNAP25 and blocks exocytosis (Fig. 3e,f)^23^,^24^, suggesting that *cis*-SNARE complexes form at boutons by exocytosis and diffuse into axons.

To test whether *trans*-SNARE complexes are converted into *cis*-SNARE complexes by exocytosis, we stimulated the boutons with a hypertonic sucrose solution, which is known to induce the exocytosis of vesicles in the readily releasable pool^25^. We found that *cis*-SNARE complexes formed in the boutons as the decay lifetime shortened there (Fig. 3g), suggesting that *cis*-SNARE complexes form from *trans*-SNARE complexes by exocytosis^1^. For time-resolved analysis, we accumulated images for 18 s from several boutons to which sucrose was applied. We therefore estimated the A1 fraction from both fitting of decay time courses and the average lifetime (*T*_A1b_; open circles), assuming a *τ*_1_ of 0.5 ns (Methods)^26^. Increases in the A1 fraction were consistently estimated by the two methods (mean±s.e. of seven experiments: 11.8±1.7 and 12.9±3.7%; Fig. 3h). This value was similar with the fraction of *trans*-SNARE complexes of 10% estimated by the subtraction method (and the gradient method, see below), suggesting that all *trans*-SNARE complexes were converted into *cis*-SNARE complexes after exocytosis. Consistent with this observation, the sucrose-induced increases in A1 values were abrogated by BoNTE treatment (Fig. 3i). These data support that *trans*-SNAREs in the boutons identified by FRET of mtq-Syx/Ven-VAMP2 actually represented the ternary complexes, and prepared for exocytosis. The A1 fractions also increased in axons after sucrose stimulation (Fig. 3g,h, *T*_A1a_), confirming the diffusion of *cis*-SNAREs into axons^13^.

Transfection of neurons with BoNTE reduced (but did not eliminate) the A1 fraction of the mtq-Syx/Ven-VAMP2 probe from 34.1 to 16.3% in boutons (Fig. 4a–c). The remaining complexes may be *trans*-SNAREs, because BoNTE nearly eliminated *cis*-SNARE (Fig. 3c), possibly by blockade of exocytosis (Fig. 4a). In fact, the BoNTE-resistant fraction (16.3%) was close to the *trans*-complex fraction estimated with the subtraction method. It is surprising that the *trans*-complex can be formed to a similar degree even without SNAP25 (Supplementary Fig. 5b), suggesting that AZ helps assemble the binary complex.

The SNARE complexes in axons are unlikely to be *trans*-complexes because vesicles are not docked in the axon. In fact, the amount of total SNARE complexes present in axons (Fig. 4b,c; 22.4%) was similar to that of *cis*-SNARE complexes (Fig. 3c,d; 21.5%) in axons. Because the fractions of *cis*-SNARE were similar between axons and boutons (Fig. 3c; 24.7%), the fraction of *trans*-SNARE complexes in boutons can be calculated by the difference between the SNARE complexes in boutons and axons as 11.7% (=34.1−22.4; Supplementary Table 1; gradient method) similar with the value (9.4%) obtained by the subtraction method. The gradient method could be applied to each bouton because the fraction of SNARE complexes in each bouton (A1b) was linearly related to that of the axon surrounding the bouton (Fig. 4d, A1a) and subtraction of A1a from A1b yielded the fraction of *trans*-SNARE in each bouton as 11.6±1.0% (*n*=36; Fig. 4e), independently of the A1a values. The sizes of boutons showed only a weak correlation with the *trans*-SNARE fractions (Fig. 4f), indicating that the sizes of boutons are not a good indicator of presynaptic function^27^, unless the sizes of AZs were taken into account (Fig. 7n).

We also found that tetrodotoxin (TTX) reduced SNARE complexes along axons within 1 h (Supplementary Fig. 7a), indicating that *cis*-SNAREs in the axons were dependent on neuronal activity, consistent with the results with BoNTE (Fig. 4c). Importantly, however, the amount of *trans*-SNARE complexes in the boutons estimated by the gradient method was unaltered up to 4 h (Supplementary Fig. 7b). This indicated that the abundant *trans*-SNAREs in the boutons were assembled in the absence of action potentials, and supported the *trans*-SNARE model for ultrafast exocytosis^5^.

### SNAP25-based FRET probes in synaptic boutons

We examined whether the fraction of *trans*-SNARE complexes obtained by SNAP25-based FRET probes was similar (Fig. 5a). The A1 fraction was 36.7% in the boutons when expression levels of VAMP2 were similar for the endogenous level on average (Fig. 5a–c; Supplementary Figs 3f and 4a). The difference between the SNARE complexes in boutons and the surrounding axons estimated *trans*-SNARE to be 14.0% (=36.7−22.7) in a bouton (Supplementary Table 1). This value was similar to those obtained using Syx-based probes (11.7%, Supplementary Table 1), suggesting that the expression levels of SNAP25 and Syx were similar in boutons. Indeed, the endogenous expression of SNAP25 and Syx was estimated to be 98 a.u. and 80 a.u., respectively, using mtq probes in boutons (Supplementary Fig. 4b,d), as in the whole brain preparation^14^. The specificity of the antibodies to Syx and SNAP25 was confirmed by transfecting the light chains of BoNTC1α-51 which selectively cleaves Syx^28^ and BoNTE (Supplementary Fig. 5b). The fraction of *trans*-SNAREs was also estimated using mtq-SNAP25b and Ven-Syx (Fig. 5f). The A1 fraction was 29.5% in boutons and 16.2% in axons for the endogenous expression level of Syx (Fig. 5g,h and Supplementary Fig. 3h), predicting that 13.3% (=29.5−16.2) exists as *trans*-SNARE complexes (Supplementary Table 1).

The A1 fractions in the boutons were reduced to 14.5% and 12.0% by BoNTC1α-51 (Fig. 5c,d), and TeNT (Fig. 5h,i)^24^, respectively. It was again noted that SNAP25/VAMP2 and SNAP25/Syx binary complexes remained in boutons, even though those in axons were eliminated (Fig. 5c,h). The fractions were similar with the estimated SNARE complex fractions of 14.0% and 13.3% (Supplementary Table 1), respectively, as in the case with BoNTE (Fig. 4c), suggesting the existence of efficient mechanisms to assemble the binary complexes in AZ.

To confirm that the FRET probes did not affect the dynamics of exocytosis and refilling of vesicles, we examined excitatory postsynaptic currents (EPSCs) evoked by electrical stimulation in the presence of cyclothiazide, which prevents the desensitization of AMPA receptors (Fig. 6a)^29^, and studied the time courses of ultrafast exocytosis, depletion and refilling of glutamatergic vesicles in wild-type (WT) mice by repetitive stimulation at 20 Hz 20 times (Fig. 6b–g). SNAP25 was replaced with mtq-SNAP25 using knockout mice^30^ where synaptic transmission was abolished (Fig. 6h,i), and rescued by mtq-SNAP25 (Fig. 6j,k). VAMP2 was then cleaved by TeNT treatment, and rescued by TeNT-resistant VAMP2 (VAMP(TeNTR)) fused with Ven (Fig. 6l–o).

In this preparation, we found the same ultrafast exocytosis as in the WT (Fig. 6a,n). EPSCs occurred with delays of about 2.5 ms, and the time to peak was 0.4 ms in the WT, SNAP25 KO with mtq-SNAP25 and SNAP25 KO with mTq-SNAP25/Ven-VAMP(TeNTR) in the presence of TeNT. Thus, the kinetics of exocytosis was unaffected. Furthermore, ultrafast exocytosis was depleted within 20 times of stimulation (Fig. 6o). This depletion lasted for at least 1 s (Fig. 6o–q), and recovered over a 10-s interval as in the WT (Fig. 6e). Thus, the depletion and refilling of vesicles were unaffected by replacing endogenous proteins with mtq-SNAP25/Ven-VAMP2. The FRET levels were similarly estimated as 24.4% in boutons and 13.1% in axons (Fig. 6r,s and Supplementary Table 1), predicting *trans*-SNAREs to be about 11.3% of the SNARE population, where the average expression levels of mtq-SNAP25 and Ven-VAMP2 were similar to endogenous levels (Supplementary Fig. 4a,d). We therefore conclude that our SNARE FRET/FLIM probes can measure the functional states of SNAREs without affecting the dynamics of SNAREs.

### Imaging of presynaptic SNARE complexes in slice cultures

We next applied our two-photon FRET/FLIM probes to the CA1 region of hippocampal slice cultures, where postsynaptic spines can be identified and the sizes of AZ can be estimated from the size of spines^15^,^26^,^31^. Fluorescence decay of mtq2-Syx^32^ was monoexponential with a time constant of 3.8 ns in the Schaffer collaterals in the CA1 region (Supplementary Fig. 8a). We first confirmed whether the *trans*-complex fraction was similar in the slice preparation by sucrose. To express the Syx-mtq2/VAMP2-Ven probes in Schaffer collateral fibres in the CA1 region, AAV1 vectors were injected into the CA3 region (Fig. 7a,b). FRET of *cis*-complexes between Syx-mtq2 and VAMP2-Ven was 3.7±1.2 % (*n*=24) in the resting state, and the A1 fraction was increased to 13.6%±1.8 % (*n*=24) during sucrose stimulation (Fig. 7c,d). The fractions of *cis*-SNARE in the resting state were similar between boutons and axons (Fig. 7e) as seen in dissociated cultures (Fig. 3d), supporting the idea that *cis*-SNAREs diffuse into axons in slice preparations (Fig. 7c), as in dissociated culture.

To measure the distribution of *trans*-SNARE complexes, we expressed mtq2-Syx/Ven-VAMP2 probes in Schaffer collateral fibres using an AAV1 (Fig. 7f), and a gene gun was used to express a plasmid containing mCherry in CA1 pyramidal neurons to visualize dendritic spines. We identified possible contacts between mtq2-positive boutons and mCherry-positive spines by three-dimensional inspection (Fig. 7g,h), where a single laser beam (900 nm) was used so that the position of two images was completely identical. We found that, in cases where presynaptic boutons overlapped with spines (Fig. 7i), mtq2-Syx showed high FRET values in the overlapping region, potentially representing AZ (Fig. 7j). Such overlaps should reflect the interdigited nature of spine synapses^33^. In fact, we also found VAMP2 clusters in such presynaptic boutons (Fig. 7k). When we analysed the fluorescent profiles along the spine (Fig. 7l), the high FRET regions were closer to the spines than to the presynaptic profiles labelled with VAMP2 or Syx, consistent with the localization of the AZ at the edge of presynaptic vesicle cluster^34^. In our expression level, mCherry fluorescence was not detectable in the mtq channel, and could not be a reason for the high FRET region at the border of boutons.

The fractions of *trans*-SNARE both in boutons on average and within AZ were estimated in the presynaptic boutons, which displayed a clear overlap with a dendritic spine (Fig. 7i). The *trans*-SNARE values were estimated by subtracting the A1 values in the axons surrounding the bouton (A1a) from those of all boutons (Fig. 7m; A1b−A1a), indicating that the average FRET values in the boutons showed a correlation with spine volume with the mean *trans*-SNARE fraction of 11.9% (Supplementary Table 1), reflecting that AZ sizes showed strong correlation with spine volumes^15^. To estimate the *trans*-SNARE fraction within AZ, we used the area of boutons overlapping with spines, and obtained the peak values (A1s) in the images which were smoothed to render the FWHM spatial resolution to 0.5 μm. The peak values of *trans*-SNARE fractions for AZ were correlated with volumes in small spines (Fig. 7n), possibly because of the limited spatial resolution, and because the fraction of *trans*-SNAREs was constant irrespective of AZ size. In fact, the peak values were independent of spine volumes for those spines >0.2 μm^3^ (Fig. 7n), where corresponding AZ had an area >0.2 μm^2^ (ref. 15) and a diameter >0.5 μm so that the peak value was not attenuated by the limited spatical resolution. For Fig. 7n, we intentionally selected large spines, as they were relatively infrequent. We estimated the fraction of *trans*-SNAREs in AZ using the spines >0.25 μm^2^ as 27.7% (=A1s–A1a; Supplementary Table 1).

To examine the presynaptic functions of spine synapses, we expressed a Ca^2+^ indicator probe, GCaMP6s, in CA1 pyramidal neurons using a gene gun for monitoring Ca^2+^ responses in individual spines (Supplementary Fig. 9a–d) whose presynaptic fibres were stimulated by a glass electrode. The release probabilities of the boutons were estimated by repetitive stimulation (20–50 times) of presynaptic fibres at 0.1 Hz, and spine Ca^2+^ increases >3 s.d. of the noise level (100% changes in GCaMP6s) within 0.3 s of stimulation were considered as successful transmission. We found that the spine size strongly correlated with the release probabilities of presynaptic terminals (Supplementary Fig. 9e), possibly owing to their correlations with the sizes of AZ^35^ and the amount of *trans*-SNARE (Fig. 7m).

### SNARE complexes in β-cells in the islets of Langerhans

Finally, we examined SNARE complexes in β cells in the islets of Langerhans (Fig. 8a), which we transfected with SNARE probes by lentivirus (Fig. 8b)^36^. We chose cells with discrete membrane staining inside the islets (Fig. 8a, arrow); 80% were β cells undergoing insulin secretion^12^,^16^. When the islets were transfected with mtq-SNAP25b and Ven-Syx (Fig. 8b), a significant A1 fraction appeared in the plasma membrane (Fig. 8c–f). The A1 value was estimated to be 5.3% at the endogenous expression level of Ven-Syx (Supplementary Fig. 10b). The existence of SNARE complexes in the islets is consistent with a previous study in which the assembly of SNAP25 with Syx was ratiometrically estimated using intramolecular FRET of SNAP25 (ref. 12). Using anti-SNARE antibodies and mtq-SNARE probes, we estimated the endogenous levels of SNAP25 and Syx expression to be 92 a.u. and 10 a.u., respectively (Supplementary Fig. 4g,i). Expression of SNAP25 was nine times greater than that of Syx, as in PC12 cells^37^ and may correlate with the small fraction of SNAP25-forming complexes with Syx.

In contrast, binding between t-SNAREs and VAMP2 was not significant (Fig. 8g–l): decay time courses were almost monoexponential (Fig. 8i,l) when islets were transfected with mtq-Syx or mtq-SNAP25b and Ven-VAMP2 (Fig. 8b). The A1 fractions were <2% and were not significantly different from zero for the endogenous expression levels of VAMP2 (Fig. 8f and Supplementary Fig. 10d,f). Thus, no significant *trans*-SNARE complexes exist in the plasma membranes of β cells (Supplementary Table 1).

**Figure 9:**
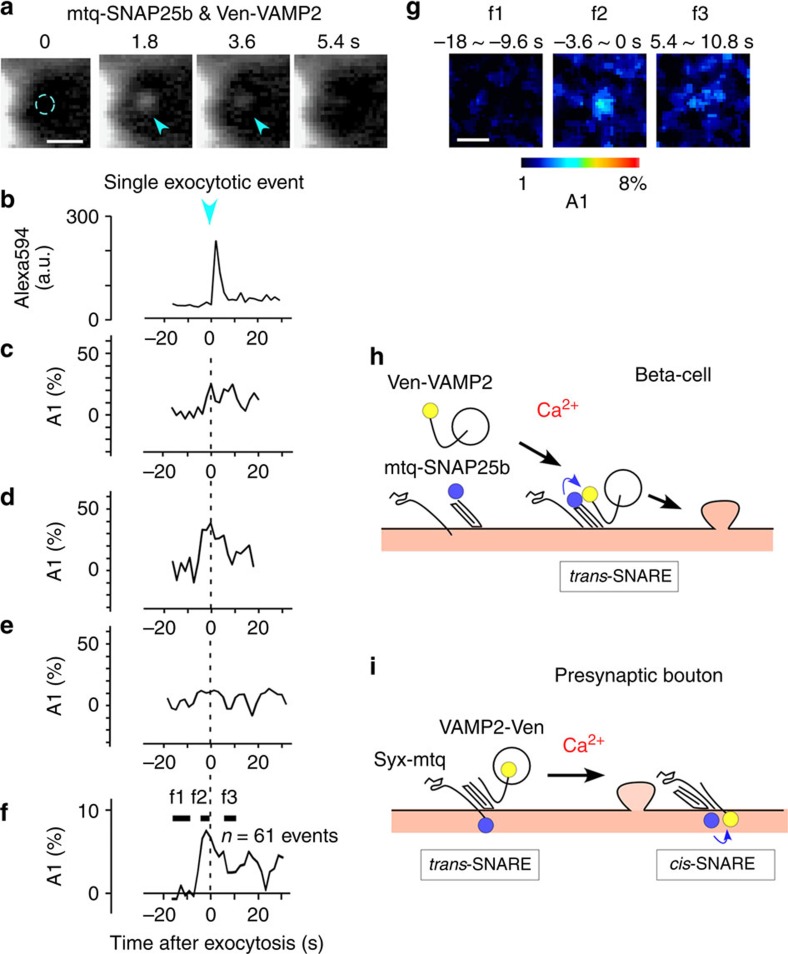
Increases in FRET signals between SNARE probes by individual exocytosis of insulin vesicles. (**a**) Fluorescence images of a pancreatic islet immersed in an Alexa594 solution, where arrowheads indicate an individual exocytotic event (scale bar, 1 μm). The islet was transfected with mtq-SNAP25 and Ven-VAMP2 using a lentivirus, and stimulated with a high-glucose (16 mM) solution. (**b**) Increases in Alexa594 fluorescence by exocytosis of a single insulin vesicle. (**c**–**e**) Increases in the binding fraction of mtq-SNAP25 and Ven-VAMP2 during individual exocytotic events. (**f**) Average time course of the binding fraction from 61 exocytotic events that were aligned to the onset of the Alexa594 signal (**b**). (**g**) A1 images before (f1 and f2) and after exocytosis (f3) (scale bar, 1 μm). (**h**,**i**) Schematic illustration of the formation of SNARE complexes and exocytosis in β cells (**h**) and presynaptic bouton (**i**).

To examine whether SNAREs probes reflected functional states in β cells, we simultaneously imaged individual instances of exocytosis of insulin vesicles with an extracellular tracer, Alexa594 (Fig. 9a,b), and FRET values of mtq-SNAP25b/Ven-VAMP2 for the region with a diameter of 0.5 μm where exocytosis was imaged during islet stimulation by a high-glucose solution (16 mM, Fig. 9c–e). We accumulated fluorescence for 1.8 s, and estimated the A1 fraction from the average lifetime, assuming a *τ*_1_ of 0.5 ns as in Fig. 3h. SNAP25b was used instead of Syx, because endogenous expression levels of Syx in β cells were low, and its overexpression was often toxic. We frequently found increases in FRET values before individual exocytotic events (1.8–10.8 s, mean=5.9±0.3 s, *n*=61). The peak value was 8.3±1.6% in averaged traces from 61 events (Fig. 9f). Such FRET signals were only found in the region surrounding exocytotic events (Fig. 9g). These data suggest that SNAREs are unassembled in the resting state, and they assemble only shortly before exocytosis in β cells (Fig. 9h), unlike synaptic vesicles in boutons where *trans*-SNAREs are already assembled in the resting state for ultrafast exocytosis (Fig. 9i).

## Discussion

We have thus succeeded in estimation of *trans*-SNARE fractions of t-SNAREs in neurons and β cells using FRET probes of SNARE proteins. We confirmed that the green fluorescent protein-fused probes could replace the functions of endogenous proteins for ultrafast exocytosis. The *trans*-SNARE fraction was consistently estimated as about 10% in the entire bouton with various probes, and about 25% in AZ, while absent from axons and β cells (Supplementary Table 1). It is of note that the gradient method with VAMP2 can estimate the fraction of *trans*-SNARE in individual boutons. This is based on the finding that most SNARE complexes in the axon were *cis*-SNAREs, and that the fractions of *cis*-SNARE were similar between boutons and their surrounding axons (Figs 3d and 7e), possibly because both *cis*-SNARE and free Syx can diffuse along axons, and their proportion is determined by a balance between exocytosis and the actions of NSF^38^,^39^. We found that the release probability is proportional to sizes of spines, and therefore to AZ sizes and to the fraction of *trans*-SNAREs. Our two-photon FRET approaches may be useful for long-term functional imaging of presynaptic terminals whose functional states cannot simply be inferred from their sizes (Fig. 4f)^27^, unlike postsynaptic dendritic spines^40^.

The fraction of VAMP2-forming complexes with Syx was estimated to be 2–5% by a previous study of presynaptic boutons^13^ in which VAMP2 was used as a FRET donor. This fraction was small because the FRET signal was diluted by VAMP2 in non-docked vesicles, the major component (90%) of vesicles in a bouton. In contrast, we used t-SNARE as FRET donor, and the FRET values are unaffected by non-docked vesicles and report the readiness of exocytosis of the plasma membrane.

We found that ∼27.7% of Syx formed *trans*-SNARE complexes in AZ (Fig. 7n). Recently, the amount of Syx1A was estimated^14^ as 20,096 molecules per bouton with an average surface area of 2.31 μm^2^. If we assume this value, the number of Syx in AZ per 0.1 μm^2^ should be estimated as 870 (=20,096 × 0.1/2.31), because Syx homogenously distribute in bouton (Figs 2a and 7g)^14^. It has been reported that the number of docked vesicles correlates with the area of the AZ, and 25 vesicles are docked per 0.1 μm^2^ of AZ^41^,^42^. By definition, the size of the AZ is nearly the same as the PSD, and the size of the PSD is proportional to the volume of the spine^15^, such that a spine with 0.1 μm^2^ PSD has 0.1 μm^3^ spine volume^15^,^27^,^41^. Thus, the number of *trans*-SNAREs associated with each docked vesicle is estimated as 9.6 (=870 × 0.277/25). Note all these *trans*-SNAREs may not be used for ultrafast exocytosis. In fact, previous studies suggested the number of functional *trans*-SNAREs required for ultrafast fusion reaction to be in between two and nine^43^,^44^,^45^,^46^.

The larger *trans*-SNARE fraction in AZ (27%) than the mean value (10%) in bouton reflects the accumulation of docked vesicles in AZ. We demonstrated that AZ promoted assembly of binary SNARE complexes (Syx/SNAP25, Syx/VAMP2, SNAP25/VAMP2) even though one type of the SNARE proteins was cleaved by clostridial toxins, possibly reflecting the fact that docking of synaptic vesicles is often unimpaired by toxin treatment^5^. An AZ protein, Munc13, may contribute in such *trans*-SNARE formation, because it induces Syx open form^47^, and its MUN domain can bind free SNARE proteins^48^,^49^. Alternatively, they may be due to tethering mechanisms, such as those involving voltage-gated calcium channels^50^.

We found that the *trans*-SNAREs were accumulated but not exclusively localized in AZ. This is consistent with the observation that docked vesicles are found outside the AZ^42^,^51^, and ectopic exocytosis of synaptic vesicles outside AZ was reported^52^,^53^,^54^. It may also relate to the fact that releasable vesicles are formed close to the Ca^2+^ channels after positional priming^55^, and that the most AZ proteins show some condensation in the AZ, but distributed throughout a bouton^14^. Such ectopic *trans*-SNAREs may be utilized for asynchronous exocytosis^56^ and tonic exocytosis^57^ where increases in [Ca^2+^]_*i*_ spread outside of AZ.

Our data indicate that a major fraction of SNARE proteins is unassembled (unitary) outside AZ even in neurons (Supplementary Table 1)^5^, and such unitary SNAREs are predominant in β cells, where *trans*-SNARE complexes are nearly absent, in line with the fact that insulin vesicles are mostly non-docked to the plasma membrane before exocytosis^5^,^58^. Indeed, we confirmed the formation of *trans*-SNARE complex shortly preceding slow insulin exocytosis (Fig. 9)^16^,^17^,^59^. Unitary SNAREs may also be utilized in neurons, for example, in slow exocytosis of large dense-core vesicles^60^.

In summary, we directly demonstrated an extreme diversity in the initial states of SNARE proteins in synapses and secretory cells, consistent with the marked differences in their readiness for exocytosis. We have thus established the technique to image the readiness for exocytosis in the plasma membrane without actually triggering exocytosis. Our two-photon FRET/FLIM methods are widely applicable for functional imaging of presynaptic terminals and secretory cells in both live and fixed preparations.

## Methods

### Ethical considerations

The use and care of animals in this study followed the guidelines of the Animal Experimental Committee of the Faculty of Medicine at the University of Tokyo.

### Construction of expression vectors

Complementary DNAs (cDNAs) of mouse SNAP25b, rat Syx and VAMP2 were kindly provided by Dr T. Abe, and mtq and Ven were provided by Dr J. Goedhart and Dr A. Miyawaki, respectively. Mammalian expression vectors for SNAREs were constructed using the ECFP-C1 or -N1 vectors (Clontech Laboratories, Inc., Mountain View, CA, USA), where fluorescence was replaced with mtq or Ven, and the promoter was switched from CMV to CAG.

To construct the N terminal fluorescent probes mtq-SNAP25 and Ven-SNAP25, SNAP25b was amplified with primers 5′-GGCGAATTCAATGGCCGAGGACGCAG-3′and 5′-CCTGTCGACTTAACCACTTCCCAGC-3′ and inserted into the EcoRI/SalI site in the multi-cloning cite of the C1 vector. To construct the double-fluorescent probe mtq-Ven-SNAP25, Ven was amplified with the primers 5′-GCCAGATCTATGGTGAGCAAGGGCGAG-3′ and 5′-GGCGAATTCGCCTTGTACAGCTCGTCCATGC-3′ and inserted into the BglII/EcoRI sites of mtq-SNAP25 in the C1 vector. To construct the N terminal fluorescent probes mtq-Syx or Ven-Syx, Syx was amplified with the primers 5′-GCAAGCTTATGAAGGACCGAACCCAGG-3′ and 5′-CGGTCGACATTCCAAAGATGCCCCCG-3′ and inserted into the Hind III/Sal I site of the C1 vector. To construct the N terminal fluorescent probes Ven-VAMP2 or mVen-VAMP2, VAMP2 was amplified with the primers 5′-GCAAGCTTGTGGTGGTTCTGGTGGTACTGGTGGTTCTGGTAT GTCGGCTACCGCTGC-3′ and 5′-CGCGGATCCCTAAGTGCTGAAGTAAACGATG-3′ and inserted into the HindIII/BamHI sites of the Ven- or mVen-containing C1 vector. TeNT-resistant VAMP2 (Q76V, F77W)^61^ was prepared with mutagenesis QuikChange II XL system, using primers 5′-CCTCCAGGCAGGGGCCTCCGTGTGGGAAACAAGTGCAGCCAAGC-3′ and 5′-GCTTGGCTGCACTTGTTTCCCACACGGAGGCCCCTGCCTGGAGG-3′.

To construct the C terminal fluorescent probe Syx-Ven, Syx was amplified with the primers 5′-GGCAAGCTTAT*CGCCACC*ATGAAGGACCGAACCCAGG-3′ and 5′-CGCGTCGACATTCCAAAGATGCCC-3′ and inserted into the HindIII/SalI sites of the N1 vector (italic sequence means Kozak (Koz) sequence). To construct the C terminal fluorescent probes VAMP2-Ven, VAMP2 was amplified with the primers 5′-GCCGCTAGC*CGCCACC*ATGTCGGCTACCGCTGCC-3′ and 5′-CGCGTCGACAGTGCTGAAGTAAACGATGATGAT-3′, and Ven was amplified with the primers 5′-GGGGTCGACGGAGGATCAGGAGGATCAGGAGGAACAGGAAT GGTGAGCAAGGGCGAGG-3′ and 5′-CGCGGATCCTTACTTGTACAGCTCGTCCATGC-3′ and the two digested PCR products were inserted into the NheI/BamHI sites of the N1 vector.

CDNAs for the light chain of BoNTE, BoNTC (51T52N53P, BoNTCα-51) and tetanus toxin (TeNT) were kindly provided by Dr H. Gaisano, Dr M. Jackson and Dr T. Abe, respectively. We constructed pCAG-Koz-BoNTE-LC-C1 by PCR amplification of the toxin with the primers 5′-GCCGGATCC*CGCCACC*ATGCCAAAAATTAA TAGTTTTAATTATAATGATCCTGTTAATG-3′ and 5′-GCCCCCGGGTTACCTTATGCCTTTTACAGAAACAATATTTTTACAAAATCTAATG-3′ and its insertion into the NheI/SmaI sites of the C1 vector by blunting. pCAG-Koz-BoNTC(51T,52N,53P)-LC-C1 was constructed by PCR amplification of the toxin with the primers 5′-GCCGCTAGC*CGCCACC*ATGCCAATAACAATTAAC AACTTTAATTATTCAGATCCTG-3′ and 5′-GCCGAATTCCTATTTATTATATAATGATCTACCATCTATTGCTTTATGACAAAATTTTGTAAATAAATAAAGC-3′ and its insertion into the NheI/EcoRI sites of the C1 vector. pcDNA3-Koz-TeNT-LC was constructed by PCR amplification of the toxin with the primers 5′-GGCGAATTC*CGCCACC*ATGCCGATCACCATCAACAACTTCC-3′ and 5′-CTTGCGGCCGCTTAAGCGGTACGGTTGTACAGG-3′ and its insertion into the EcoRI/NotI sites of the C1 vector. All cDNA constructs were confirmed by DNA sequencing.

### Dissociated cultured cortical neurons

Neocortical neurons were dissociated from male Sprague-Dawley rat brains (E19) or C57BL/6 mouse brains (E17.5) with 0.15% trypsin in ethylenediaminetetraacetic acid (Invitrogen, Carlsbad, CA, USA) and DNase I (30 μg ml^−1^). The cells were plated on Laminin- and poly-D-lysine-coated cover glasses at a density of 1.7 × 10^5^ cells per cm^2^. The neurons were maintained in a serum-free medium (Neurobasal Medium; Invitrogen) supplemented with 500 μM glutamine (rat) or 1% GlutaMax (mouse) and 2% B-27 Supplement (Invitrogen). Plasmids for the FRET probes and light-chain clostridial toxins were transfected by lipofection with Lipofectamine 2000 (Invitrogen; 0.25%) according to the manufacturer's protocol at 10–12 days after culture, and the cells were observed 1–2 days after lipofection with 0.8–1.2 μg DNA. The standard external bathing solution (SolA) contained 150 mM NaCl, 5 mM KCl, 1 mM MgCl_2_, 2 mM CaCl_2_, 10 mM 4-(2-hydroxyethyl)-1-piperazineethanesulfonic acid-NaOH (pH 7.4) and 10 mM glucose for neurons or 2.8 mM glucose for islets (300–310 mOsm). The sucrose solution for stimulating presynaptic terminals contained 300 mM sucrose, and its osmolarity was 610 mOsm l^−1^.

SNAP25 KO mice were kindly provided from Wilson MC, Takahashi M, Yamamori S. Genotype was defined by PCR using cerebellar DNA and PrimeSTAR system (TaKaRa Bio). Primer mixtures were NEO588-Forward primer (5′-GCCGAATATCATGGTGGAAA-3′), 25KO-57849FL (5′-CCCACATCTGCCTCCTTTCTGGTT-3′) and 25KO-58090RL (5′-GGGATGGGCACCATCAAATCTTTTC-3′, 20 μM each). 240 bp band was detected with WT, while 530 bp band was detected with hetero or KO. Lentivirus carrying mtq-SN25 and Ven-VAMP2 (TeNT-resistant) was prepared^62^, and their promoter regions were substituted into CaMKII promoter. Lentivirus carrying mtq-SN25 (1–3 × 10^11^ genome copies per μl) was transfected in the first day of culture to rescue survival, and lentivirus carrying Ven-VAMP2 (TeNT resistant) was transfected 2 days before electrophysiological experiments. Tetanus toxin (Lubio Science, 50 ng ml^−1^) was added 24–36 h before experiments. TTX (10 μM) was added 1–4 h before recordings.

### Electrophysiological experiments

The postsynaptic cells (DIV 12–16) were whole-cell clamped (holding potential=−70 mV) and perfused with intracellular solution (in mM: CsCl 140, HEPES 10, MgATP 4, Na_2_GTP 0.3, Na_2_-phophocreatine 10, Alexa594 0.025, pH=7.25 with CsOH). Theta-burst stimulation was applied (20 Hz × 20 pulse, 100 mA amplitude, 0.1 ms duration, 100–150 μm from dendrites) with 1- or 10-s interval. Extracellular solution was based on SolA containing 10 mM CaCl_2_, 100 μM cyclothiazide, 50 μM picrotoxin, 320 nM Na-pyruvate and pH 7.4.

### Experiments with hippocampal slices

For slice-cultured neurons, the expression vector was prepared by inserting double-floxed inverted-orientation (DIO) mtq2-Syx, Syx-mtq2, Ven-VAMP2 or VAMP2-Ven into the pAAV vector under the control of the human Syn (hSyn) promoter. The mtq2 (ref. 32) cDNA was acquired from pCAG-mTq-C1 plasmid by mutagenesis. Briefly, an I146F mutation was introduced by PCR with primers (5′-AACTACTTTAGCGACAACGTCTATATC-3′, 5′-GTCGCTAAAGTAGTTGTACTCCAGCTT-3′) using PrimeSTAR Max mutagenesis kit (Takara, Japan). The product was digested by Nhe I and BsrG I and was subcloned to the AAV vector. Recombinant AAV1 vectors were produced using previously described methods^63^. In brief, the expression vectors were transfected into AAV293 cells with pHelper (Stratagene, La Jolla, CA, USA) and pRep-Cap AAV1 (Applied Viromics LLC, Fremont, CA, USA) and the cell lysate was purified by CsCl gradient, dialysed and concentrated using Amicon Ultra filters (EMD Millipore, Billerica, MA, USA). Viral titre was determined by quantitative reverse transcription (qRT)–PCR using primers designed to detect the promoter regions. Hippocampal slices (350-μm thick) were prepared at P7 from sd rat, mounted on 0.4 μm culture plate inserts (EMD Millipore) and incubated at 35 °C in an atmosphere of 5% CO_2_ in medium comprising 50% minimum essential medium (Invitrogen), 25% Hanks' balanced salt solution (Invitrogen), 25% horse serum (Invitrogen) and 6.5 g l^−1^ glucose. AAV mixture (for example, AAV-hSyn-DIO-mTq2-Syx1A, AAV-hSyn-DIO-Ven-VAMP2 and cre expression vector; AAV1. CAMKII 0.4 Cre.SV40 (Penn Vector Core, Philadelphia, PA USA)) was injected into CA3 pyramidal cell layer at P8–9, using glass micropipette (diameter ∼6 μm). The mixture also contained 0.03% Fast Green. Transfection of pCAG-mCherry was done by Gene gun at P14–19 (3.5 μg per 30 μl gold) to visualize postsynaptic dendrite. In the electrophysiological experiments, slices were individually transferred to recording chambers and superfused with artificial cerebral spinal fluid containing 125 mM NaCl, 2.5 mM KCl, 2 mM CaCl_2_, 1 mM MgCl_2_, 1.25 mM NaH_2_PO_4_, 26 mM NaHCO_3,_ 200 μM Trolox (Sigma-Aldrich Corporation, St Louis, MO, USA), 5 nM TTX (Wako, Osaka, Japan) and 20 mM glucose bubbled with 95% O_2_ and 5% CO_2_. All physiological experiments were performed at 32–34 °C. For the imaging of cellular structures and Ca^2+^ transients evoked by electrical stimulation of presynaptic fibres, neurons were transfected with mCherry and GCaMP6s (Plasmid #40753, Addgene, Cambridge, MA, USA), respectively, using a gene gun (PDS-1000; Bio-Rad Laboratories, Inc., Hercules, CA, USA) at P14 and used for experiments between P17 and P22.

### Culture and gene transfer with pancreatic islets

Pancreatic islets were isolated from male imprinting control region mice (>8-weeks old) by collagenase digestion (Sigma-Aldrich Corporation) as described previously^59^. The islets were infected with a lentiviral vector carrying cDNA of the fluorescent probes for 24–72 h in Dulbecco's modified Eagle's medium (glucose 1 g l^−1^) supplemented with 10% foetal bovine serum, 100 U ml^−1^ penicillin and 100 mg ml^−1^ streptomycin^36^. Before imaging, the islets were transferred on to thin (0.1 mm) glass coverslips (Matsunami Glass Ind., Ltd., Osaka, Japan) in the recording chamber. As for the lentiviral vector system, in brief, cDNAs of fluorescent SNAREs were inserted into the FCMV-WPRE vector, and virus particles were produced by their cotransfection with pCMV-VSV-G and pCMV-dR8.9 into lenti-X cells. The viruses were purified by ultracentrifugation and their titre was checked using an qRT–PCR titration kit (6 × 10^9^ genome copies per ml; Lenti-X; Clontech Laboratories, Inc.).

### Immunostaining

For immunostaining, neurons in dissociated culture and pancreatic islets were washed with phosphate-buffered saline (PBS), fixed with 4% paraformaldehyde for 1 h (4 °C), washed with PBS, permeabilized with 0.3% Triton X-100 for 5 h and blocked with 2.5% horse serum and 0.15% Triton X-100 for 8 h. The primary antibodies, anti-SNAP25 polyclonal antibody (SNAP25 specific and unable to bind SNAP23; immunogen: 195–206 AA; ab5666; Abcam, Cambridge, UK; 1:100), anti-VAMP2 rabbit polyclonal antibody (VAMP2-specific and unable to bind VAMP1, 3, 4 and 8; immunogen: 1–18 AA; ab3347; Abcam; 1:1000), anti-Syx antibody (also possibly binds Syx1B; ab41453; 1:200) and anti-synaptophysin antibody (MAB5258; EMD Millipore; 1:200), were applied for 8 h, following which the samples were washed with PBS and the secondary antibody was applied (Alexa-Fluor 594-labelled goat anti-mouse Immunoglobulin G (H+L); Invitrogen; 1:800) for 8 h. After a final wash, the preparations immersed in PBS were observed using two-photon microscopy. Fluorescence intensities of Alexa-Fluor 594 were quantified in many plasma membrane regions in the pancreatic islets or in the presynaptic terminal regions of neurons.

The intrinsic expression of SNAREs was measured by Alexa-Fluor 594 intensity after secondary antibody labelling. The expression levels of mtq- or Ven-labelled SNAREs were compared with endogenous expression, taking into account the reduction in the fluorescent intensity of mtq and Ven resulting from fixation to 50.2% and 31%, respectively (Supplementary Fig. 4).

### Two-photon excitation FLIM

Two-photon excitation imaging was performed using an inverted laser-scanning microscope (FV1000 and IX81; Olympus Corporation, Tokyo, Japan) equipped with a water-immersion objective lens (UPlanApo60xW/IR; numerical aperture 1.2; Olympus Corporation) and a femtosecond laser (Mai Tai; Spectra Physics Inc., Mountain View, CA, USA) for dissociated culture and β cells in the islets. For slice experiments, an upright microscope (BX61WI; Olympus Corporation) with a LUMPlanFL60xW objective (numerical aperture 0.9; Olympus Corporation) was used. Fluorescence of Alexa-Fluor 594, Ven and mtq was excited at 830 nm and detected at 570–650 nm, 515–560 nm and 460–500 nm, respectively. The autofluorescence was <2% of mtq fluorescence in our expression levels, and could not contribute to the lifetime measurement. To measure Ven intensity, the excitation wavelength was set at 970 nm. We confirmed that a spectral bleed through of Ven into the mtq channel was undetectable in our expression levels irrespective of the excitation wavelength of 830 or 970 nm even after repeated imaging sessions. Laser power was set at 4.5 mW. Pixel size was 0.10 μm and 512 × 512 pixels, with a frame rate of 1 frame per 6 s. Ten frames were accumulated for the analysis, except for Fig. 3g,h, where six and three frames were used, respectively. For the acceptor bleaching experiment (Supplementary Fig. 2a), dissociated culture preparations were irradiated with a Hg lamp at wavelength of 500–550 nm for about 40 min.

FLIM of dissociated culture and β cells in the islets was performed using a GaAsP avalanche-diode hybrid detector (R10467U-40; Hamamatsu Photonics K. K., Hamamatsu, Japan) and analysed using SPCImage software (SPC-150; Becker & Hickl GmbH, Berlin, Germany). For hippocampal slice preparations, we used GaAsP PMT (H7422; Hamamatsu Photonics K. K.) and analysed data using PicoHarp (PicoQuanta GmbH, Berlin) and homemade MATLAB software (MathWorks, Inc., Natick, MA, USA). The fluorescence decay time course fitted a double-exponential function, with time constants of 0.5 and 3.5 ns for mtq, which were convoluted with a Gaussian system response function with a s.d. of 0.1 ns. The A1 values were not critically dependent on the fast time constant between 0.5 and 1 ns. The fast time constant was well-predicted by the short linkers between SNAREs and mtq (or Ven) of 18–25 amino acids in our constructs with predicted lengths of 2.3–2.7 nm, assuming that the length of flexible linker was estimated as 0.86 nm per 2.4 amino acids^64^, and that the physical length depends on the square root of the number of amino acids^65^,^66^. Comparable lengths of linkers were used for FRET/FLIM probes, and resulted in similar fast lifetime decays in other studies^26^,^67^.

Because the fluorescence decay of mtq was 3.5 ns (*τ*_2_) and it did not decay completely within the 12.5-ns sampling interval (*T*), *A*_*i*_ obtained from double-exponential fitting was multiplied by (1-e^−*T*/^*τ*^i^), and then normalized by *A*_1_+*A*_2_=1 (incomplete multi-exponential fitting)^68^. This process is simple, and the usage of fluorescent probe with a slow fluorescent lifetime decay is beneficial for a better separation of the fast FRET component. The A1 fractions were pseudocolour coded, and the luminosities of the colour code were modulated on the basis of the brightness of mtq. In the time-lapse experiments (Fig. 3e–h), each image of A1 was calculated with a detection time of 20 s and filtered by a median filter with a size of 2 × 2 pixels. In the time-lapse experiments, the A1 fractions were obtained in two different ways. One way was the curve fitting of the average of data from many boutons (Fig. 3f,h; filled symbols). The other way was to use the average lifetime (Fig. 3h, open symbols) to calculate A1, assuming that *τ*_1_ and *τ*_2_ were constantly 0.5 and 3.5 ns and using the equations 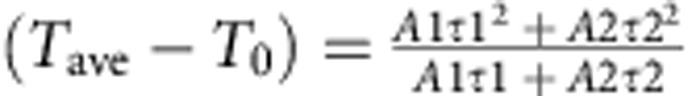 and *A1*+*A2*=1, where *T*_ave_ is the average lifetime and *T*_0_ is the offset arrival time, obtained by fitting the fluorescence of a large region-of-interest^10^.

To measure mtq and mCherry in hippocampal slice preparations, the excitation wavelength was set at 900 nm and fluorescence was detected at 460–500 nm and 570–650 nm, respectively. Laser power was set at 5–8 mW. Pixel size was 0.079 μm (128 × 128 pixels). Three-dimensional FLIM images were obtained by 20 s imaging for each depth with 1-μm interval (seven depths). The Ven images were acquired at 520–560 nm at 980 nm excitation wavelength, and Z-stacks had an interval of 1 μm with a frame rate of 1 frame per 0.8 s. We estimated spine-head volume (*V*) from the total fluorescence intensity (*F*) in the stacked images of spines. A conversion coefficient, *V*/*F*, was obtained from the fluorescence profile of the largest, roundest spine on each dendrite^69^,^70^, which was subsequently applied to all other spines on the dendrite.

For Ca^2+^ imaging, two-photon excitation was performed at 970 nm and the fluorescence intensity of GCaMP6s was measured at 490–540 nm. The signals were obtained from all spines. Pixel size was 0.1 μm, and images were acquired at a rate of 1 frame per 0.22 s. Two-photon images were analysed using IPLab (Scanalytics Inc., Milwaukee, WI, USA), ImageJ (the National Institutes of Health, Bethesda, MD, USA; http://rsb.info.nih.gov/ij/) and MATLAB software.

Detection of insulin exocytic events was performed as described in a previous paper^12^. Shortly, we immersed the pancreatic islets expressing the FRET probes with SolA containing 0.3 mM Alexa594 hydrazide (Invitrogen), and stimulated insulin secretion with 16 mM glucose and 5 nM GLP-1. The individual exocytic events were captured with 2P imaging by the sudden appearance of small fluorescent spots inside the islet^16^. We simultaneously performed time-lapsed measurements of Alexa 594 intensity at 570–650 nm (Fluoview) and FLIM of mtq at 460–500 nm with an interval of 1.8 s. We used excitation wavelength of 830 nm, and the power of 4–5 mW. Exocytic events detected within 40 min were analysed.

### Statistical analysis

The FRET images which did not have sufficient photon counts were discarded from statistical analysis. Comparable numbers of images were used as in previous studies^8^,^12^,^13^. Histogram bars represent the mean±s.e.m. The significance of each A1 fraction versus 0% was tested using the Wilcoxon signed-rank test (Figs 1, 2, 3, 4, 5, 6, 7, 8 and Supplementary Fig. 5). The differences between two groups were tested using the Mann–Whitney *U*-test (Figs 2, 3, 4 and Supplementary Figs 5, 7). For multiple comparisons between groups, the significance was first examined using the Kruskal–Wallis test, followed by Scheffé's or Steel's tests (Figs 2, 5 and 8). The significances of the correlation coefficient were tested using Spearman's rank correlation coefficient (Figs 3, 4 and 7, Supplementary Figs 3, 9 and 10). No randomization and blinding was used to analyse the data.

## Additional information

**How to cite this article:** Takahashi, N. *et al*. Two-photon fluorescence lifetime imaging of primed SNARE complexes in presynaptic terminals and β cells. *Nat. Commun*. 6:8531 doi: 10.1038/ncomms9531 (2015).

## Supplementary Material

Supplementary Information Supplementary Figures 1-10 and Supplementary Table 1
