# Supplementary material for: Two-photon fluorescence lifetime imaging of primed SNARE complexes in presynaptic terminals and β cells
Source: Nat Commun. 2015 Oct 6;6:8531. doi: 10.1038/ncomms9531 (PMC4600761; doi:10.1038/ncomms9531)
Supplement: Supplementary Information — Supplementary Figures 1-10 and Supplementary Table 1 [file ncomms9531-s1.pdf]

# Supplementary Figure 1

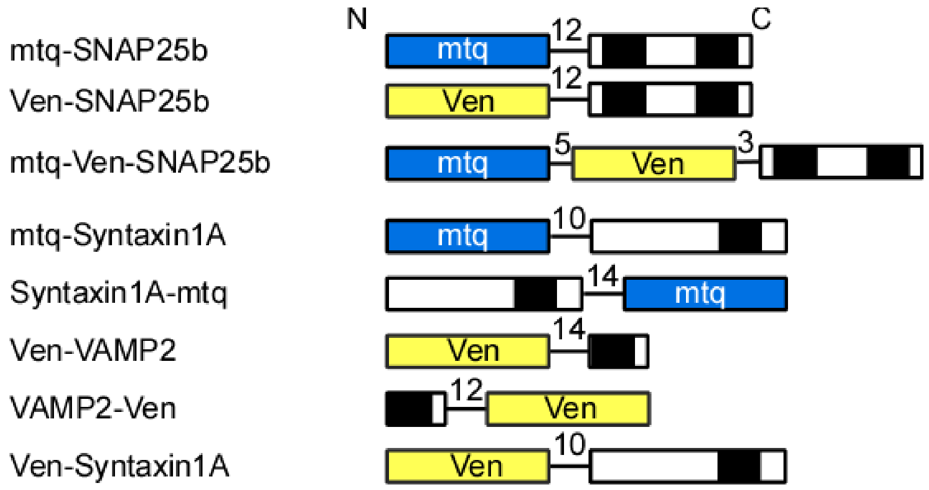


**Supplementary Figure 1 | SNARE Probes for FRET/2pFLIM Analysis Used in the Present Study.** mTurquoise (mtq) and Venus (Ven) are in blue and yellow, respectively. The soluble *N*-ethylmaleimide-sensitive factor attachment protein receptor (SNARE) motifs are in black. The lengths of the linker amino acids between the SNARE motives are indicated.

# Supplementary Figure 2

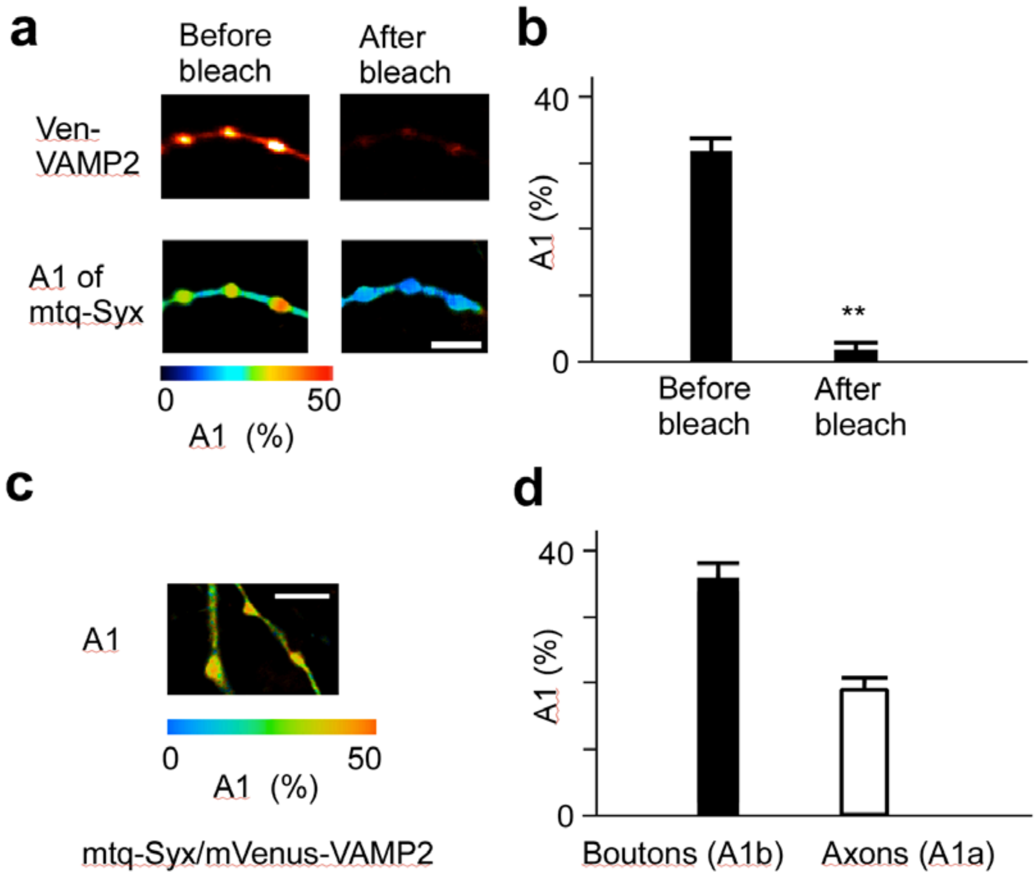


**Supplementary Figure 2 | FRET from mtq-Syx to Ven-VAMP2 detected by 2pFLIM.**

(**a**) Images of Ven fluorescence and the A1 fraction of mtq-Syx before and after photobleaching of Ven in the axonal boutons (scale bar , 5 μm). (**b**) The A1 fractions of boutons before photobleaching (31.7 ± 1.9%, 20 boutons) for endogenous expression levels of Ven-VAMP2 expression (mean ± SD = 712 ± 111 A.U.) and after photobleaching (1.8 ± 1.1%) of Ven-VAMP2 (mean ± SD = 198 ± 22 A.U). **p = 0.0004 with the Mann–Whitney *U*-test. (**c**) An image of the A1 values of mtq-Syx for mVenus-VAMP2 (scale bar , 5 μm). (**d**) The A1 values obtained by mtq-Syx/mVenus-VAMP2 in bouton (36.3 ± 2.2%, 12 boutons) with the expression level of mVenus of 46-185% endogenous level (mean ± SD = 587 ± 69 A.U.) and in axon (19.4 ± 1.5%, 11 axons), which are not significantly different from those obtained by mtq-Syx/Ven-VAMP2 (Fig. 2i; p = 0.56 and 0.25 in boutons and axons, respectively).

## Supplementary Figure 3

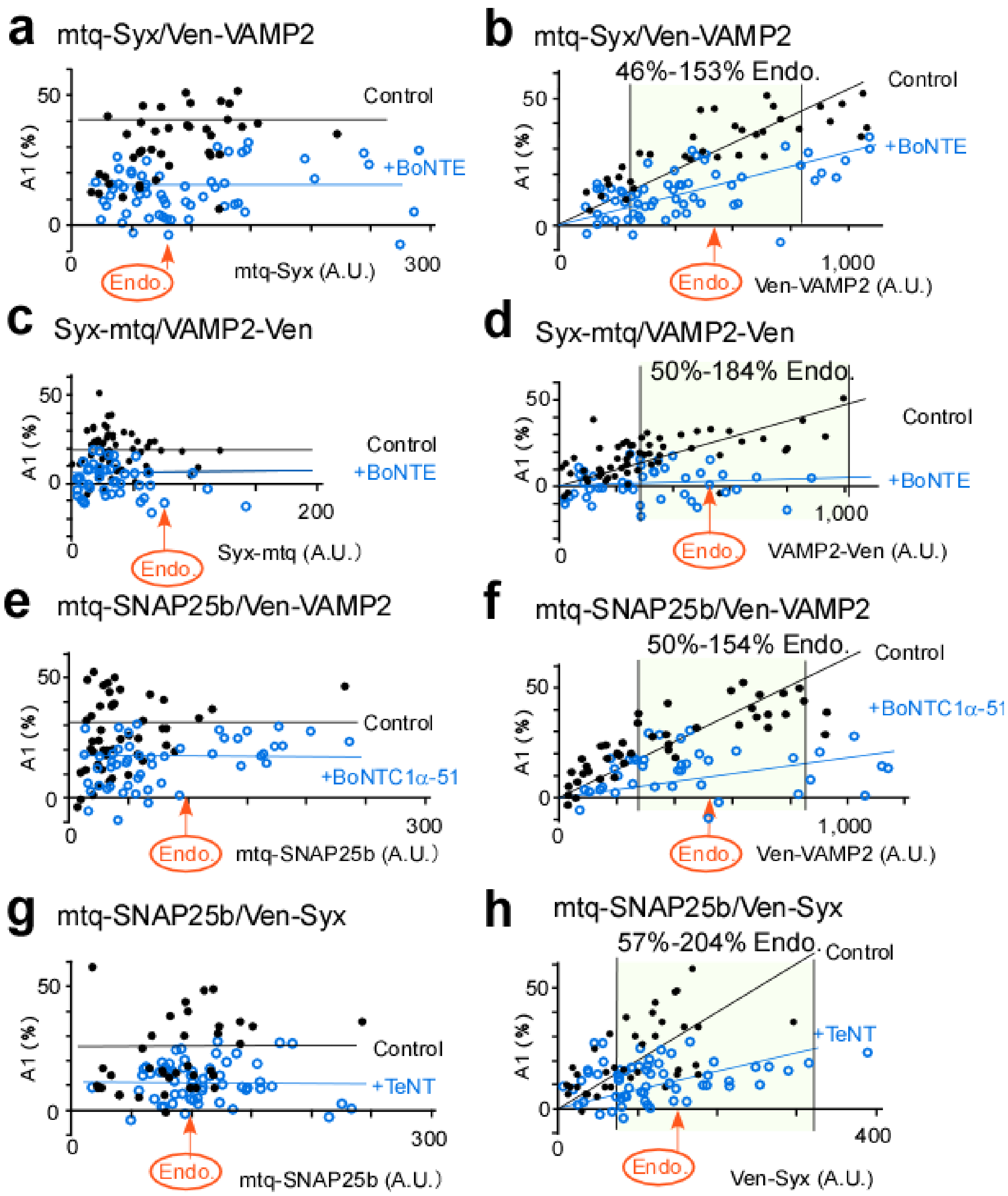


**Supplementary Figure 3 | The Dependence of the A1 Fractions on the Expression Levels of FRET Acceptors in the Neurons.**

The A1 values for mtq-Syx/Ven-VAMP2 (**a** and **b**), Syx-mtq/ VAMP2-Ven (**c** and **d**), mtq-SNAP25/Ven-VAMP2 (**e** and **f**) and mtq-SNAP25/Ven-Syx (**g** and **h**) plotted against the expression levels of three SNAREs measured by mean fluorescence intensity (arbitrary unit) of each probe in each bouton in the absence and presence of clostridial

toxins. The endogenous expression levels of each SNARE protein, obtained from Supplementary Fig. 4, are indicated with red ovals denoted as ‘Endo.’. The A1 values shown in **Figs. 2–5** are estimated from the data depicted in the green shaded area so that the averaged values were similar to the endognenous level of 540 A.U. for VAMP2 and 48 A.U. for Syx: 46%–153% (**b**, mean ± SD = 546 ± 38 A.U., *n* = 21 and 516 ± 40 A.U., *n* = 39, for control and BoNTE), 50%–184% (**d**, mean ± SD = 524 ± 45 A.U., *n* = 24 and 508 ± 22 A.U., *n* = 20, for control and BoNTE), 50%–154% (**f**, mean ± SD = 549 ± 36 A.U., *n* = 20 and 551 ± 45 A.U., *n* = 24 in control and BoNTC1α-51), 57–204% (**h**, mean ± SD = 138 ± 10 A.U., *n* = 21 and 139 ± 9, *n* = 45 in control and TeNT). The solid lines are horizontal in (**a**, **c**, **e** and **g**) and are through the origin with the least square fits of the data (**b**, **d**, **f** and **h**). The correlations were infrequently significant with the donor expression. The correlation coefficients were 0.44, -0.08, 0.24 and 0.34 for 38, 60, 47 and 37 control boutons, and p values were 0.002, 0.75, 0.28 and 0.16 in (**a**), (**c**), (**e**) and (**g**), respectively, by Spearman’s rank correlation coefficient. In contrast, the A1 values showed more correlation with the acceptor expression. The correlation coefficients were 0.73, 0.61, 0.78 and 0.59 for 38, 60, 47 and 37 control boutons, 0.60, 0.07, 0.33 and 0.26 for toxin treated preparations, and the p values were 0.0000, 0.0000, 0.0000 and 0.0000 for control and 0.16, 0.07, 0.0016, 0.06 for toxin treated preparations in (**b**), (**d**), (**f**) and (**h**), respectively.

# Supplementary Figure 4

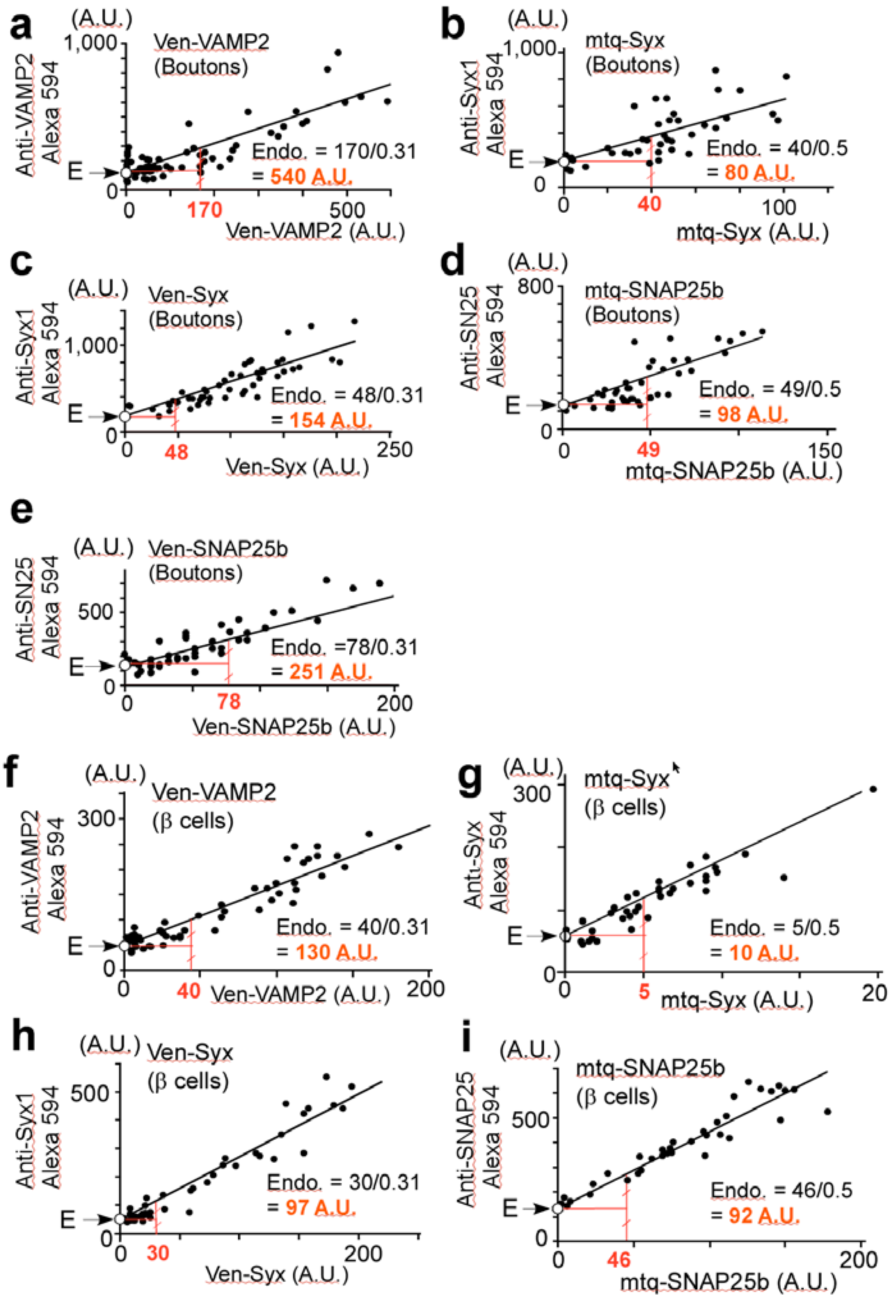


**Supplementary Figure 4 | Endogenous Expression Levels of VAMP2, syntaxin1A (Syx) and SNAP25.**

Fluorescence intensities of boutons stained with anti-SNARE antibodies are plotted against the fluorescence intensity of the same proteins labelled either with Venus (Ven) or mTurquoise (mtq) for boutons in rat dissociated culture (**a–e;** *n* = 69, 51, 51, 46, 43) or β cells in the islets of Langerhans (**f–i**; *n* = 55, 37, 44, 35). The endogenous level of

Alexa-Fluor® 594 staining with anti-SNARE antibodies was obtained from the cells not expressing the FRET probes (arrows labelled ‘E’). In non-transfected boutons, Alexa-Fluor® 594 fluorescence intensities were 123 ± 14 arbitrary units (A.U.; mean±standard error, 14 boutons) for VAMP2 (**a**), 183 ± 7 A.U. (32 boutons) for Syx (**b** and **c**), 135 ± 9 A.U. (37 boutons) for SNAP25 (**d** and **e**). For non-transfected β cells, Alexa-Fluor® 594 fluorescence intensities were 49 ± 4 A.U. (14 cells) for VAMP2 (**f**), 53 ± 3 A.U. (32 cells) for Syx (**g** and **h**) and 129 ± 4 A.U. (9 cells) for SNAP25 (**i**). The solid lines represent the least square fit. The same levels of expression were attained at the levels of expression of fluorescent SNAREs, as indicated by ‘Endo.’. The endogenous expression levels (A.U.) of three SNAREs were estimated by dividing the values with 0.31 or 0.5 for Ven and mtq, as indicated, because fixation of the preparations reduced the intensity of fluorescence of these chromophores to 31% ($n$ = 37) and 50.2% ($n$ = 40), respectively.

# Supplementary Figure 5

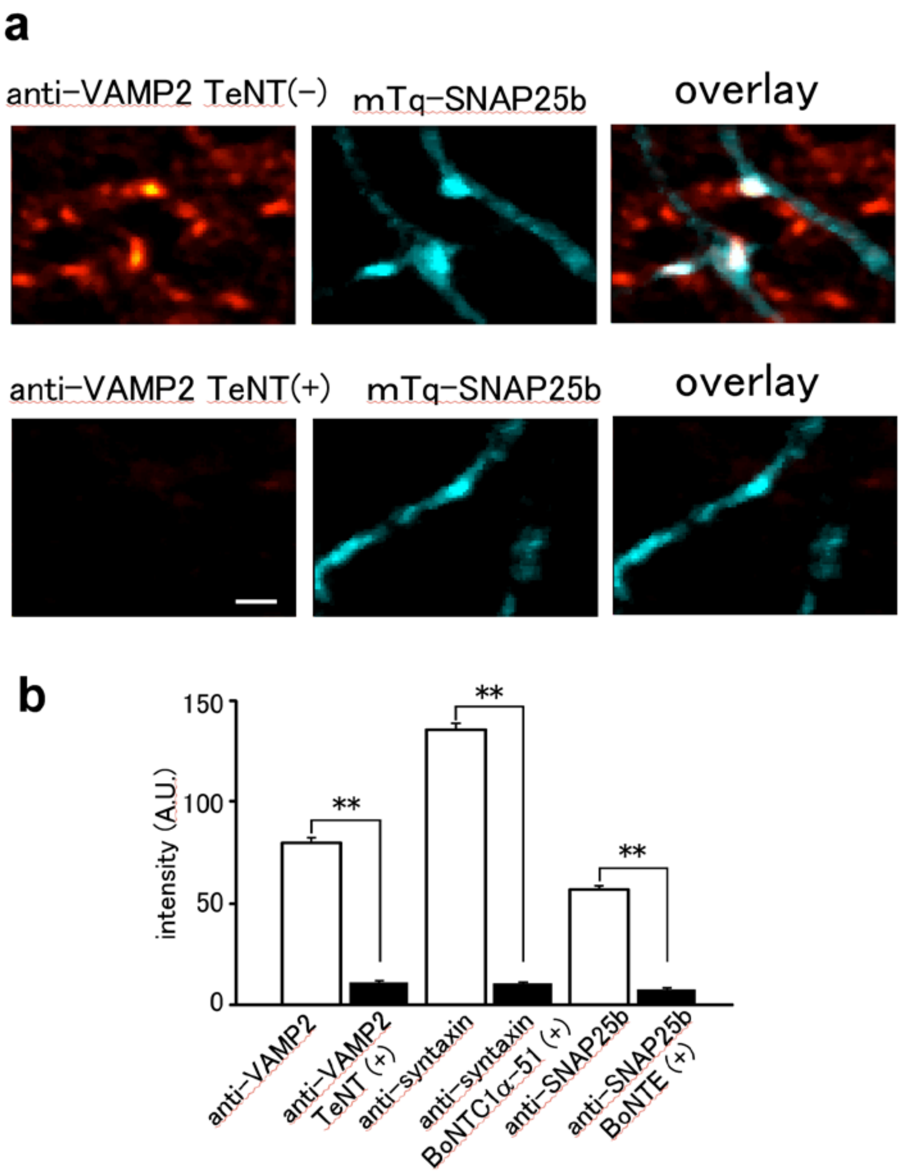


**Supplementary Figure 5｜Specificity of antibodyes against VAMP2, Syx and SNAP25.**

(**a**) Images of anti-VAMP2 conjugated with Alexa594, mtq-SNAP25b and their overlays in dissociated culture preparations without and with expression of the light chain of TeNT (scale bar, 2 μm). (**b**) Alexa fluorescence intensities conjugated with anti-VAMP2 antibody in control (80.3 ± 2.6 A.U., 20 boutons) and in TeNT (11.1 ± 0.79 A.U., 20 boutons), with anti-Syx antibody (135.7 ± 3.4 A.U., 20 boutons) and in BoNTC1α-51 (10.6 ± 1.1 A.U., 20 boutons), and with anti-SNAP25b (57.0 ± 2.1 A.U., 20 boutons) and in BoNTE (7.4 ± 1.1 A.U., 20 boutons). **$p < 0.001$ with the Mann–Whitney *U*-test.

# Supplementary Figure 6

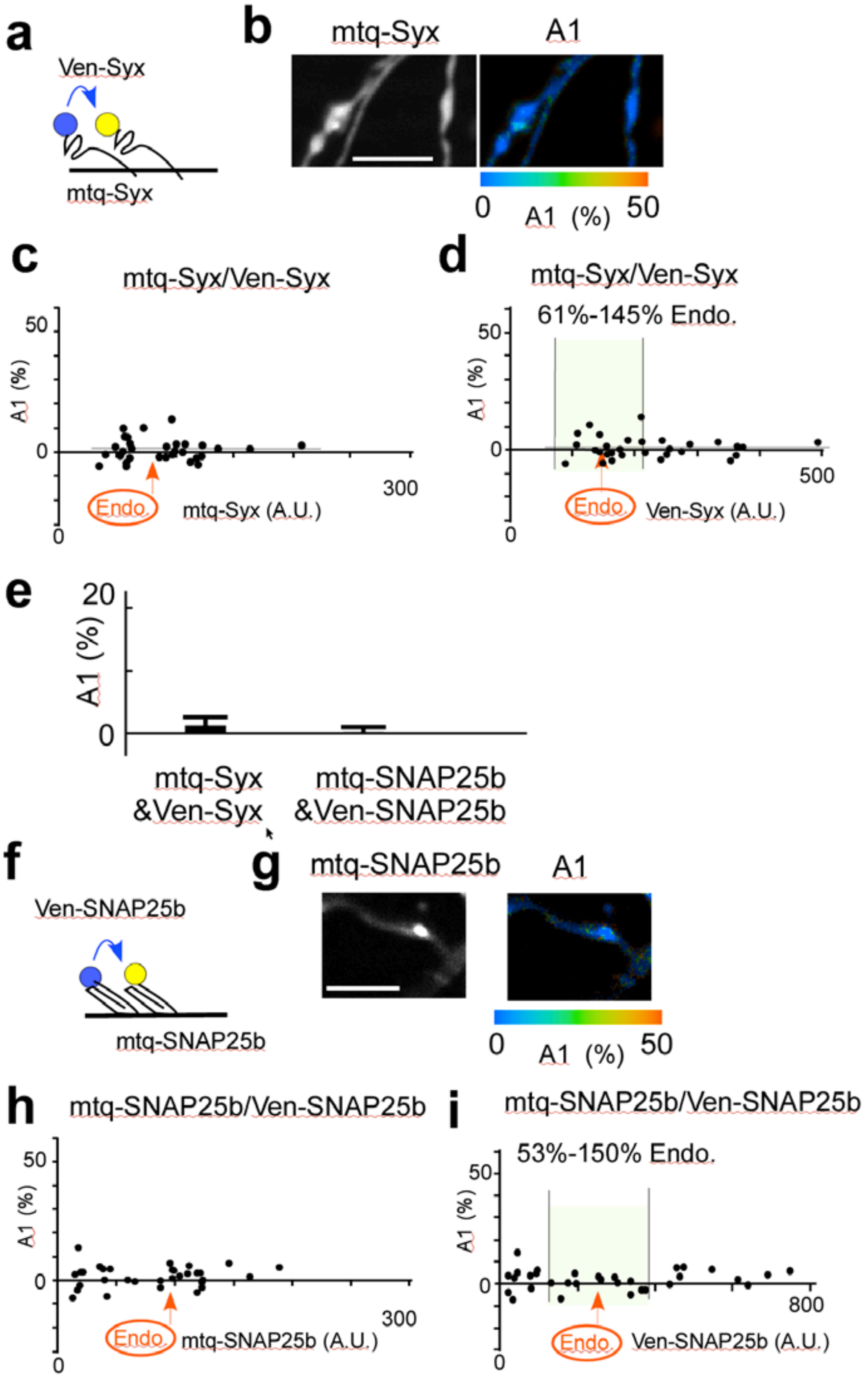


**Supplementary Figure 6 | FRET/2pFLIM analysis of homotypic Syx or SNAP25 binding.**

(**a**) Schematic drawing of mtq-Syx/Ven-Syx. (**b**) Fluorescence and A1 images of mtq-Syx/Ven-Syx-expressing boutons (scale bar, 5 μm). (**c** and **d**) The A1 values among

boutons with various expression levels of Syx and SNAP25. The green shaded area was used to obtain the A1 value for the endogenous expression level of the acceptor Syx of 145 A.U.: 61%–145% (**d**, mean ± SEM = 148 ± 8 A.U., *n* = 16). (**e**) The A1 values of mtq-Syx/Ven-Syx (1.2 ± 1.4%, *n* = 16) and mtq-SNAP25b/Ven-SNAP25b (1.4 ± 1.1%, *n* = 13) with the endogenous expression levels of Syx and SNAP25, respectively (Supplementary Fig. 4d,i). Wilcoxon signed-rank test versus 0%: p = 0.63 and p = 0.17, respectively. (**f**) Schematic drawing of mtq-SNAP25/Venus-SNAP25. (**g**) Fluorescence and A1 images of mtq-SNAP25/Ven-SNAP25-expressing boutons (scale bar, 5 μm). (**h** and **i**) The A1 values among boutons with different expression levels of mtq-SNAP25b (**h**) or Ven-SNAP25b (**i**). The green shaded area was used to obtain the A1 value for the endogenous expression level of the acceptor SNAP25 of 156 A.U.: 50%–167% (**i**, mean ± SD = 158 ± 20 A.U., *n* = 15).

## Supplementary Figure 7

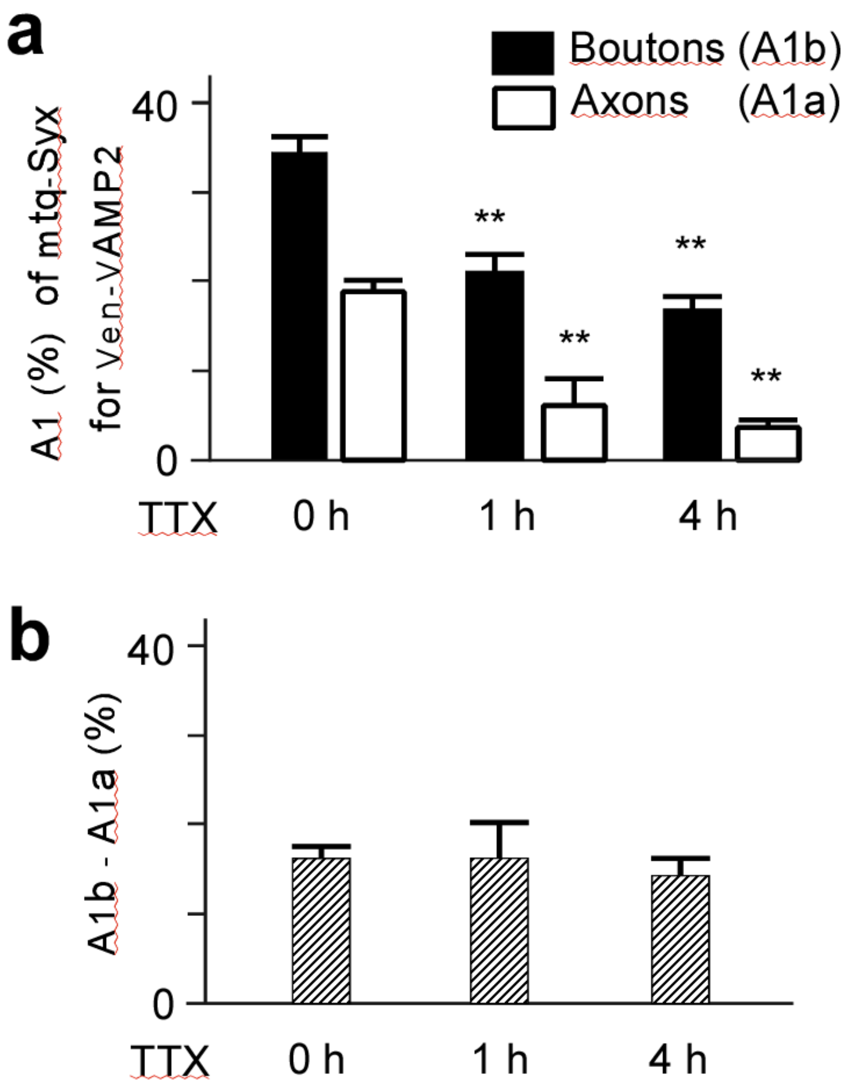


**Supplementary Figure 7 | TTX effects on the A1 fractions for mtq-Syx/Ven-VAMP2.**

(**a**) The A1 fractions (34.5 ± 1.8%, 16 boutons; 18.9 ± 1.8%, 21 axons) in control, and those (20.9 ± 2.1%, 21 boutons; 6.1 ± 3.0%, 12 axons) 1 h after treatment of TTX, and those (16.9 ± 1.4%, 23 boutons; 3.6 ± 0.9%, 10 axons) 4h after treatment of TTX. **$p < 0.001$ with the Mann–Whitney *U*-test vs. 0 h. (**b**) The *trans*-SNARE fractions obtained by the gradient method in control (16.0 ± 1.2%, 15 boutons), 1 h after TTX treatment (16.0 ± 4.2%, 11 boutons) and 4 h after (14.1 ± 1.9%, 10 boutons).

# Supplementary Figure 8

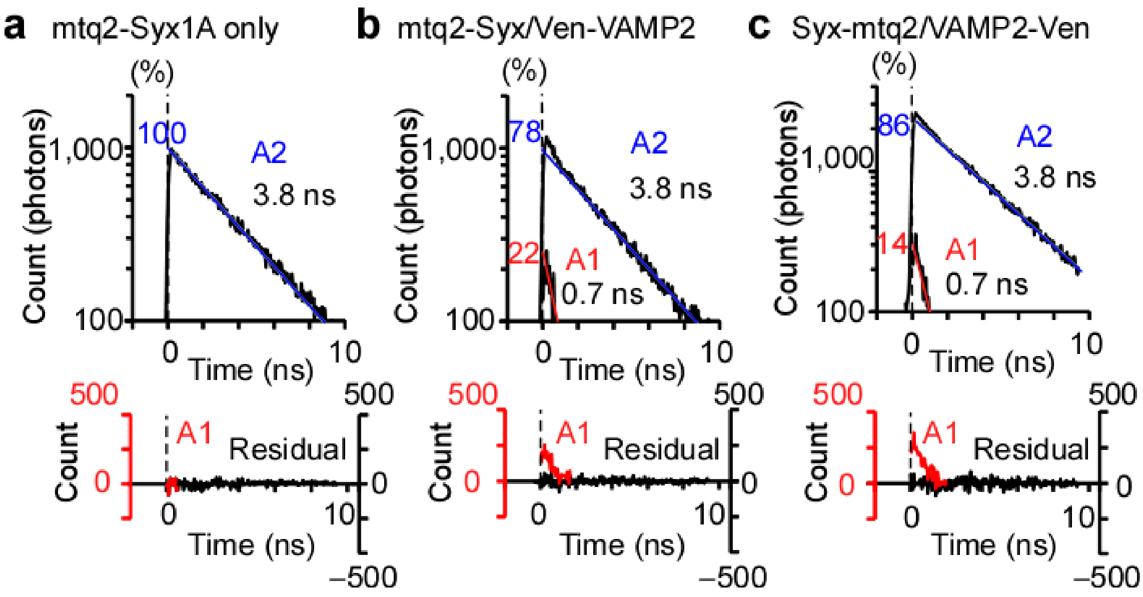


**Supplementary Figure 8 | Lifetime decay curves in boutons of CA1 pyramidal neurons in hippocampal slice cultures.**

Slice preparations were transfected with mtq2-Syx (**a**); mtq2-Syx/Ven-VAMP2 (**b**); Syx-mtq/VAMP2-Ven (**c**) expressed using AAV1/2 virus-mediated gene transfer into the CA3 region, and lifetime decay were analyzed as in Figure 2F and H. The fluorescence data were accumulated from 8, 38, and 24 boutons. $\tau_2$ was set as 3.8 ns in **(a)**, (**b**), and (**c**).

# Supplementary Figure 9

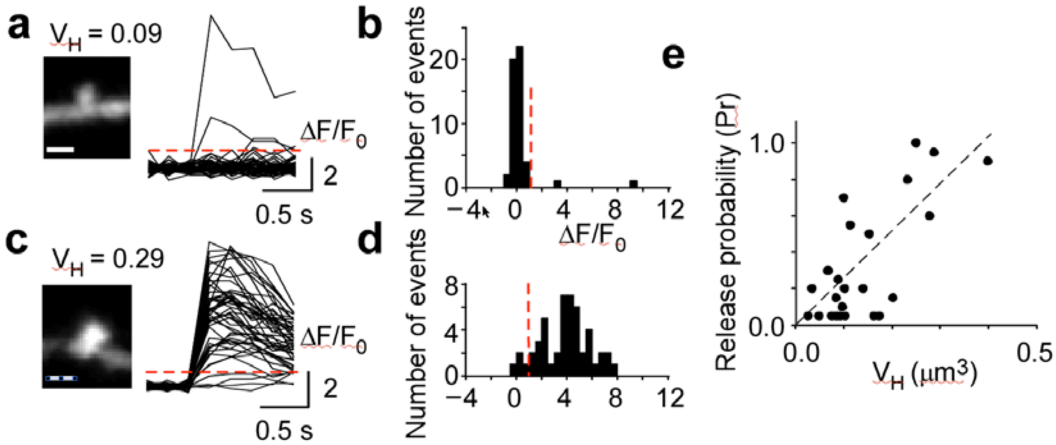


**Supplementary Figure 9 | $Ca^{2+}$ transients in dendritic spines of CA1 pyramidal neurons evoked by electrical stimulation of presynaptic fibers in hippocampal slice cultures.**
(**a** and **c**) Images of spines (single Z-section) from which $Ca^{2+}$ imaging was performed with GCaMP6s during repetitive presynaptic stimulation, as indicated in the right panels (scale bars, 1 μm). (**b** and **d**) Amplitude histograms of evoked $Ca^{2+}$ responses. The red lines indicate the threshold $Ca^{2+}$ responses for successful release events, which are defined as 100% increases in GCaMP signals above three times the standard deviation of the baseline noise. (**e**) Probability of success rate plotted against spine volume ($p = 0.0062$ using Spearman's rank correlation coefficient; $r = 0.73$; $n = 25$).

## Supplementary Figure 10

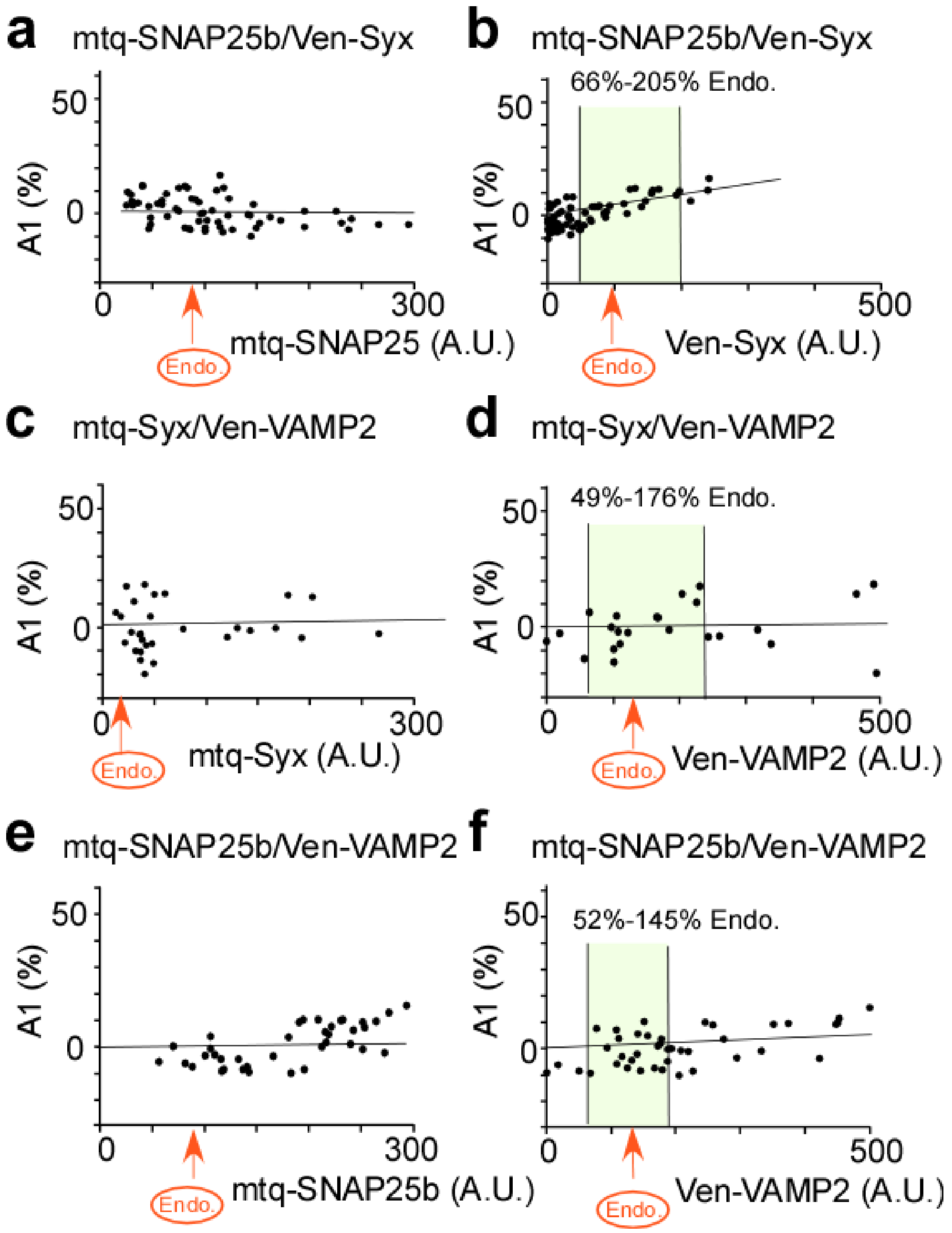


**Supplementary Figure 10 │ The Dependence of the A1 Values on the Expression Levels of the Acceptors in β cells in the Pancreatic Islets.**

The A1 values for mtq-SNAP25/ Ven-Syx (**a** and **b**), mtq-Syx/Ven-VAMP2 (**c** and **d**) and mtq-SNAP25/Ven-VAMP2 (**e** and **f**) plotted against the expression levels of three SNAREs measured by fluorescence intensity of each probe in the region of interest encompassing the plasma membrane in arbitrary unit. The endogenous expression levels

of each SNARE protein, 97A.U. and 130A.U. for SNAP25 and VAMP2. The A1 values shown in Figure 8 are estimated from the data using the expression levels of the donor indicated by green shaded areas: 66%–205% (**b**, mean ± SEM = 114 ± 9 A.U., $n$ = 22), 49%–176% (**d**, 139 ± 15 A.U., $n$ = 15) and 52–145% (**f**, 138 ± 8A.U., $n$ = 21). The solid lines are horizontal in (**a**, **c**, **e** and **g**), and through the origin with the least square fits of the data (**b**, **d**, **f** and **h**). The correlation coefficients were −0.1, 0.15 and 0.75 for 68, 29 and 39 islet cells, respectively, and the p values were 0.001, 0.75 and 0.0001 in (**a**), (**c**) and (**e**), respectively, using Spearman's rank correlation coefficient. In contrast, the A1 values were more correlated with acceptor expression, particularly in (**b**). The correlation coefficients were 0.74, 0.21 and 0.5 for 68, 29 and 39 islet cells, respectively, and the p values were 0.0001, 0.28 and 0.0002 for (**b**), (**d**) and (**f**) respectively.

## Supplementary Table 1

| Preparations | Methods | *Trans*-SNARE fractions |
|---|---|---|
| Dissociated culture | "Subtraction Method" | Mean ± SEM |
| | Syx/VAMP2 in axons | 0.9% ± 2.2% |
| | Syx/VAMP2 in boutons | 9.4% ± 3.6% |
| | "Gradient Method" in boutons | |
| | Syx/VAMP2 | 11.7% ± 3.0% |
| | SNAP25/VAMP2 | 14.0% ± 2.5% |
| | SNAP25/VAMP2 in KO mice | 11.3% ± 1.7% |
| | SNAP25/Syx | 13.3% ± 3.4% |
| | Syx/VAMP2 in AZ | 24.2% ± 2.7% |
| Slice culture | "Gradient Method" in boutons | |
| | Syx/VAMP2 | 11.9% ± 10.2% |
| | Syx/VAMP2 in AZ | 27.7% ± 2.1% |
| Pancreatic islet β-cells | Total SNARE complexes in the plasma membrane | |
| | Syx/VAMP2 | 0.8% ± 2.3% |
| | SNAP25/VAMP2 | 0.04% ± 1.4% |

**Supplementary Table 1 | Estimated fractions of *trans*-SNARE complexes in Syx or SNAP25.** Three different preparations, cortical neurons in dissociated culture, hippocampal pyramidal neurons in slice cultures and β-cells in the islets of Langerhans. "Subtraction method" utilized the FRET values estimated from the total SNARE assembly minus *cis*-SNARE assembly using Syx/VAMP2 (**Figs. 2–4**). "Gradient method" can be applied to all three pairs of SNAREs, and the fractions in the axons were subtracted from those in the boutons (**Figs. 3–5,7**). *Trans*-SNARE in the active zone was estimated using the peak A1 values in dissociated culture (**Fig. 2j**), and those of presynaptic boutons facing the spine with volume larger than 0.25 $\mu m^3$ (**Fig. 7n**). The total binary SNARE complexes were estimated in the plasma membranes of β-cells (**Fig. 8**).
